# Well‐being decline during adolescence: school transition as a predominant driver beyond age progression

**DOI:** 10.1111/jcpp.70154

**Published:** 2026-04-21

**Authors:** Mi Zhou, Carol Maher, Sally Brinkman, Juliette Cools, Dorothea Dumuid

**Affiliations:** ^1^ Alliance for Research in Exercise, Nutrition and Activity Adelaide University Adelaide South Australia Australia; ^2^ College of Education, Behavioural and Social Sciences Adelaide University Adelaide South Australia Australia; ^3^ South Australia Department for Education Adelaide South Australia Australia

**Keywords:** Primary‐to‐secondary school transition, well‐being, student, moderation analysis, longitudinal analysis

## Abstract

**Background:**

The transition from primary‐to‐secondary school significantly impacts students' well‐being. However, existing research provides limited insight into the long‐term impact of school transition on well‐being, and no studies have disentangled age‐related changes from transition‐specific effects. This study leverages a natural experiment, where an educational reform resulted in two different age cohorts transitioning simultaneously, to disentangle age effects from transition effects on student well‐being.

**Methods:**

This study analyzed longitudinal data from the Well‐being and Engagement Collection census (2019–2025) in South Australia. Participants were two cohorts of students who simultaneously started secondary school in 2022: one transitioning at Year 7 and the other at Year 8. Well‐being was measured across eight domains. Linear mixed‐effects regression models examined transition effects and tested interactions with sociodemographic factors.

**Results:**

A total of 20,910 participants (Male: 52.1%, Age in 2019: 9.7 ± 0.6) contributing 104,800 observations (5.0 responses/participant) across the 7 years were included. In the first 2 years post‐transition, well‐being experienced adverse changes across all domains (marginal effects for positively‐worded measures: −0.44 to −0.18; negatively‐worded measures: 0.08 to 0.13). The largest declines were observed in cognitive engagement (−0.44) and perseverance (−0.31). Younger and older cohorts experienced similar adverse changes; however, the younger cohort showed a larger well‐being decline in the second‐year post‐transition. Females experienced more pronounced declines than males. The well‐being decline among students residing in remote and very remote areas persisted until the third year after the transition.

**Conclusions:**

School transitions negatively affect students' well‐being, with impacts that persist for more than 2 years. This decline was largely attributable to the school transition rather than age‐related progression. Females and students residing in remote areas experienced greater declines in well‐being than their counterparts. These findings highlight the need for transition‐specific support strategies for vulnerable groups that extend beyond the first year of secondary schooling.

## Introduction

The transition from primary‐to‐secondary educational institutions, known as the primary‐to‐secondary school transition, has been widely recognized as a significant and challenging process for students (Hopwood, Hay, & Dyment, 2016; Kaur, McLoughlin, & Grimes, 2022; Zeedyk et al., 2003). During this transition, students encounter various challenges, including adapting to an unfamiliar physical environment, leaving close friends and familiar adults behind, establishing new friendships, navigating different social groups, and meeting more demanding academic requirements (Symonds, 2015). Prior research has shown that the transition to secondary school can have a substantial impact on students' social and emotional well‐being. It can lead to increased anxiety (Hannah & Topping, 2012; Waters, Lester, Wenden, & Cross, 2012), depression (Waters et al., 2012), and decreased self‐efficacy, school belongingness, and connectedness (Lofgran, Smith, & Whiting, 2015; Vaz et al., 2014; Witherspoon & Ennett, 2011).

According to life course theory, experiences during key developmental transitions can have enduring effects on individuals' later development and well‐being, particularly when these transitions involve changes across multiple life domains (Benner & Mistry, 2020; Elder Jr, 1998). The transition from primary‐to‐secondary school typically coincides with early adolescence. Disruptions or challenges experienced during this transition may therefore be amplified and alter well‐being trajectories in ways that extend beyond short‐term adjustment, shaping experiences across secondary school and potentially into adulthood (Eccles, Lord, & Buchanan, 2018). However, most existing studies have used cross‐sectional or two‐wave longitudinal designs to compare well‐being solely between the final year of primary school and the first year of secondary school. Only a limited number of studies have adopted multi‐year approaches to examine the long‐term transition impact on well‐being (Jindal‐Snape & Cantali, 2019; West, Sweeting, & Young, 2010).

Moreover, the causal relationship between school transitions and the decline in well‐being has not been firmly established (Jindal‐Snape, Hannah, Cantali, Barlow, & MacGillivray, 2020; West et al., 2010). In fact, well‐being often tends to decline as students grow older (Blanchflower & Oswald, 2008). Attributing this decline in well‐being mainly to the effects of this school transition could potentially result in an overestimation of its impact. Yet, there are no studies that examine how much the decline in well‐being during the transition deviates from the preceding age‐related trajectory.

A unique educational reform in South Australia provides an opportunity to disentangle the effects of age from those of the school transition. In 2022, the state of South Australia shifted its secondary school entry point from Year 8 (age 13–14) to Year 7 (age 12–13), resulting in two different age cohorts transitioning simultaneously. This natural experiment allows us to examine whether transition effects on well‐being vary by age, addressing a key gap in the literature.

This study aims to describe the longitudinal trajectories of student well‐being before and after the primary‐to‐secondary school transition and, using a natural experiment, to disentangle age‐related changes from transition effects. As the impact of the transition varied across students from different sociodemographic backgrounds, this study also examines the moderating roles of sex, language background, regional location, and parental education level. The specific research questions are “How does student well‐being change over time across the primary‐to‐secondary school transition?” “Is the observed decline in student well‐being during the transition to secondary school due to age‐related development or the transition itself?” and “Are transition‐related changes in student well‐being moderated by sex, language background, regional location, and parental education level?”

## Methods

### Study design and participants

The Well‐being and Engagement Collection (WEC) is a state‐wide census designed to collect data on non‐academic factors that are pertinent to the overall well‐being of students in Year 4 through to Year 12 in South Australia. The census was conducted by the South Australian Department for Education (DfE) annually during Weeks 2 to 5 of the second school term (May). All government schools in South Australia were invited to participate. The DfE assigned participants unique identifiers, enabling the linkage of individual‐level longitudinal data across timepoints. This project received an ethics exemption from the Human Research Ethics Committee of the University of South Australia (application #202625). The data were accessed through an approved application to the South Australian DfE. All datasets were de‐identified prior to release and stored on password‐protected servers, with access restricted to authorized members of the research team in accordance with Departmental data governance and data handling requirements. The results of this study were reported in accordance with the Strengthening the Reporting of Observational Studies in Epidemiology (STROBE) guidelines.

All WEC participants who commenced secondary school in 2022 were included. Due to educational reforms introducing an earlier transition, two cohorts of students transitioned to secondary school simultaneously in 2022: those moving into Year 7 (Younger) and those moving into Year 8 (Older).

### Well‐being measures

Eight WEC well‐being domains (Cognitive engagement, Happiness, Optimism, Perseverance, Emotional regulation, Life satisfaction, Sadness, and Worry) were included for analysis. These domains were developed based on established measures and validated in Australian samples, with internal consistency (Cronbach's *α*) ranging from 0.63 to 0.93 (Gregory et al., 2019, 2022; Gullone & Taffe, 2012; Kern, Benson, Steinberg, & Steinberg, 2016). Participants rated each item on a 5‐point Likert scale ranging from 1 (strongly disagree/almost never/not at all like me) to 5 (strongly agree/almost always/very much like me) (Gregory et al., 2019). Domain scores (range 1–5) were computed as the mean of items within each domain. Higher scores reflected better well‐being for positively‐worded domains (Cognitive engagement, Happiness, Optimism, Perseverance, Emotional regulation, Satisfaction with life), whereas higher scores indicated worse well‐being for Sadness and Worry. The definitions of well‐being domains are presented in Table S1.

### Exposure

Seven waves of data (2019–2025) were included to capture students' well‐being trajectories across the transition from primary‐to‐secondary school. In Australia, the school year begins in late January; thus, the 2021 assessment corresponds to the final year of primary school (pre‐transition), whereas the 2022 assessment reflects the first year of secondary school (post‐transition). Accordingly, 2019 and 2020 represent 2 and 1 years before the transition, while 2023 to 2025 correspond to the second to fourth years following the transition.

**Figure 1:**
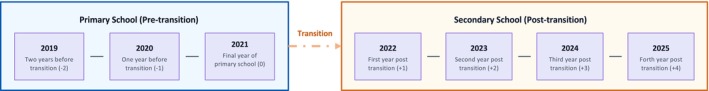
Exposure timeline

The variable years since transition was created to quantify the time elapsed since the transition, coded as follows: −2 for the 2 years before the transition (2019), −1 for the 1 year before the transition (2020), 0 for the final year of primary school (2021), and +1 to +4 for 1 to 4 years after the transition (2022–2025). This variable was treated as the exposure in subsequent analyses. The full exposure timeline is illustrated in Figure 1.

### Covariate

Sociodemographic information, including sex, primary language spoken at home, residential region, and highest parental education level, was obtained from school enrolment data reported by parents or caregivers prior to the start of each academic year. The information was administratively linked with WEC data. Participants reporting a gender other than male or female (alternative response options were only available in 2020) were excluded because the data were not consistently available across waves. Accordingly, our findings are reported by sex rather than by gender. The primary language spoken at home was categorized as “English” or “Language other than English (LOTE).” The highest parental education level refers to the highest attainment by either parent and was classified into three categories: year 12 or less (including no school qualifications, year 9 or less, year 10, year 11, and year 12), diploma (certificate I to IV, advanced diploma/diploma), and bachelor degree or above. The variable was coded using the highest level reported across all available time points. Residential region was classified based on the postcode of main residence, using the Australian Bureau of Statistics Accessibility and Remoteness Index of Australia (Australian Bureau of Statistics, 2021) into major city, inner and outer regional, and remote and very remote. This classification is based on road distance to service centers of varying population sizes, reflecting differences in access to services, infrastructure, and population centers across Australia. Due to the potential confounding of a physical change in school environment at the transition, an additional variable was created to describe whether students changed schools during the primary–secondary school transition, as some schools have co‐located primary and secondary campuses. Responses were coded as “Yes” if students changed schools and “No” if they remained in the same school.

### Statistical methods

Linear mixed‐effects regression models were employed with years since transition as the exposure variable and the eight WEC well‐being outcomes as dependent variables (eight models, one model per outcome). School and individual levels were considered as independent random intercepts to account for clustering of participants within schools and repeated observations within individuals. Students' sex, residential region, highest parental education level, primary language spoken at home, and change in physical school environment were included as covariates in the regression models. This process was repeated for the interaction models *Years since transition* × age cohort (young/old) and *Years since transition* × Sociodemographics. Multivariate *F* tests were performed to estimate the global effect of time on well‐being measures. Model‐estimated marginal means [95% CI] at each calendar year were calculated and visualized to facilitate visual comparisons. To explore whether well‐being differed between consecutive years, marginal effects [95% CI] were also calculated.

All analyses were conducted in R statistical software (version 4.3.1), using the lme4, ggplot, and marginaleffects packages (Arel‐Bundock, Greifer, & Heiss, 2024; Bates, Mächler, Bolker, & Walker, 2015; Villanueva & Chen, 2019).

## Results

### Participant characteristics

The characteristics of participants are shown in Table 1. A total of 20,910 participants contributing 104,800 observations were included in this study. On average, each individual student completed 5.0 annual surveys. Baseline characteristics were comparable between included and excluded participants. The participant flowchart was shown in Figure S1. The characteristics of participants across calendar year and age cohort were reported in Tables S2 and S3, respectively.

**Table 1:** Characteristics of participants.

Characteristics	Levels	Included (*N* = 104,800)	Excluded (*N* = 29,391)
Age (years)	Mean ± SD	12.6 ± 2.0	12.3 ± 2.3
Sex, *n* (%)	Female	50,248 (47.9%)	14,277 (48.6%)
	Male	54,552 (52.1%)	15,101 (51.4%)
	Missing	/	3
Primary language spoken at home, *n* (%)	English	80,972 (77.3%)	19,369 (65.9%)
	Not English	23,828 (22.7%)	10,022 (34.1%)
Residential region, *n* (%)	Major city	73,981 (70.6%)	21,904 (74.8%)
	Inner and outer regional	26,923 (25.7%)	6,480 (22.1%)
	Remote and very remote regional	3,896 (3.7%)	916 (3.1%)
	Missing	/	81
Highest parental education level, *n* (%)	Year 12 or less	18,863 (18.0%)	4,458 (18.4%)
	Diploma	50,378 (48.1%)	9,413 (38.8%)
	Bachelor degree or above	35,559 (33.9%)	10,410 (42.9%)
	Missing	/	5,100
Change in physical school environment during the transition, *n* (%)	No	10,860 (10.4%)	546 (20.5%)
	Yes	93,940 (89.6%)	2,112 (79.5%)
	Missing	/	26,723
Age cohort, *n* (%)	Old	51,823 (49.4%)	14,036 (47.8%)
	Young	52,977 (50.6%)	15,345 (52.2%)
Calendar year, *n* (%)	2019	14,820 (14.1%)	6,260 (21.3%)
	2020	13,761 (13.1%)	5,320 (18.1%)
	2021	17,761 (16.9%)	4,747 (16.2%)
	2022	16,101 (15.4%)	2,289 (7.8%)
	2023	15,496 (14.8%)	2,933 (10.0%)
	2024	14,183 (13.5%)	3,623 (12.3%)
	2025	12,678 (12.1%)	4,209 (14.3%)
Grade, *n* (%)	4	7,261 (6.9%)	3,137 (10.7%)
	5	14,544 (13.9%)	5,864 (20.0%)
	6	15,670 (15.0%)	5,465 (18.6%)
	7	16,849 (16.1%)	3,058 (10.4%)
	8	15,998 (15.3%)	2,614 (8.9%)
	9	14,912 (14.2%)	3,225 (11.0%)
	10	13,569 (12.9%)	3,857 (13.1%)
	11	5,997 (5.7%)	2,161 (7.4%)
Well‐being domain			
Cognitive engagement	Mean ± SD	3.6 ± 0.8	3.7 ± 0.8
	Missing	4,712	1,692
Happiness	Mean ± SD	3.7 ± 0.8	3.8 ± 0.8
	Missing	1,436	749
Optimism	Mean ± SD	3.6 ± 0.9	3.7 ± 0.9
	Missing	1,908	858
Perseverance	Mean ± SD	3.6 ± 0.8	3.7 ± 0.8
	Missing	1,701	881
Emotional regulation	Mean ± SD	3.3 ± 0.9	3.4 ± 0.9
	Missing	2,386	1,038
Satisfaction with life	Mean ± SD	3.5 ± 0.9	3.6 ± 0.9
	Missing	2,004	928
Sadness	Mean ± SD	2.9 ± 1.0	2.8 ± 1.0
	Missing	2,325	938
Worry	Mean ± SD	3.1 ± 1.0	3.1 ± 1.0
	Missing	2,462	1,005

### Well‐being changes during the school transition

**Figure 2:**
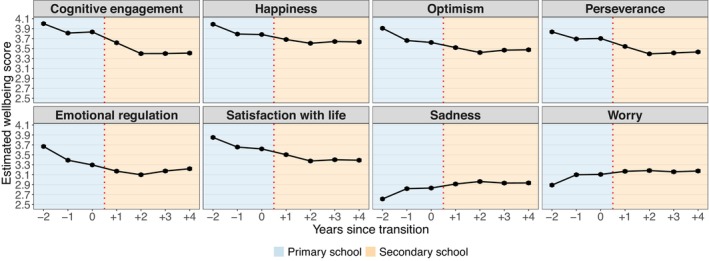
Well‐being trajectories during the transition in South Australia

**Figure 3:**
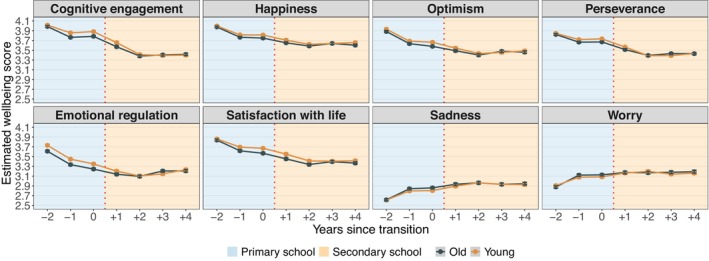
Well‐being trajectories during the transition across age cohorts in South Australia

**Figure 4:**
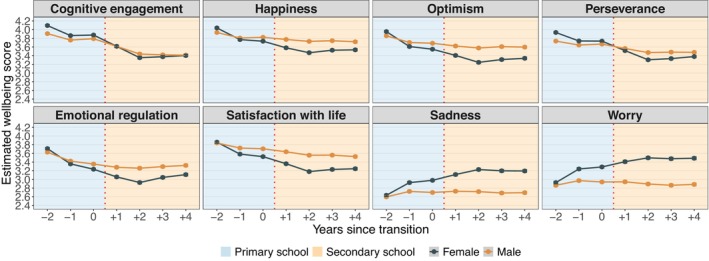
Well‐being trajectories during the transition across sex in South Australia

**Figure 5:**
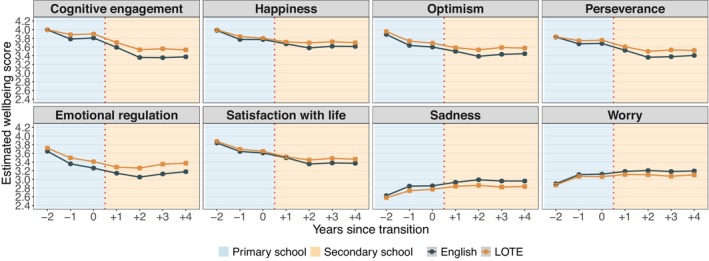
Well‐being trajectories during the transition across primary language spoken at home in South Australia

**Figure 6:**
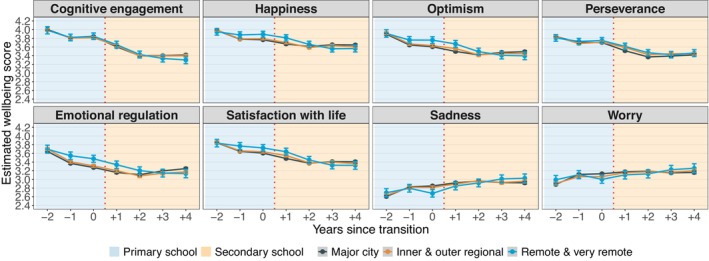
Well‐being trajectories during the transition across residential region in South Australia

**Figure 7:**
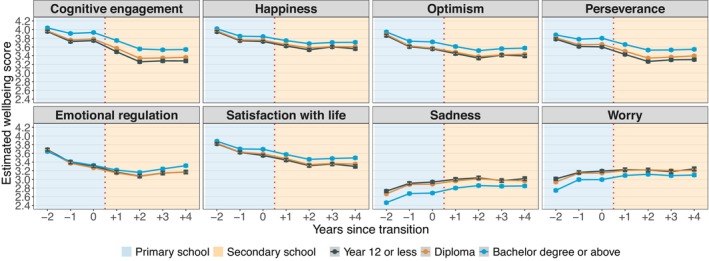
Well‐being trajectories during the transition across highest parental education level in South Australia

Figures 2, 3, 4, 5, 6, 7 illustrate the trajectories of estimated well‐being over time. The shaded block in blue represents the primary school period, and the shaded block in yellow represents the secondary school period. Statistically significant changes were observed across all well‐being measures over time (all *p <* .001, Table S4). Detailed marginal means are presented in Tables S5–S10. Marginal effects are provided in Tables S11–S16.

Figure 2 illustrates well‐being trajectories for all participants. During the pandemic period (−2 ~ −1), students experienced a sharp decline in well‐being across all domains [Marginal effects for positively‐worded measures (Cognitive engagement, Happiness, Optimism, Perseverance, Emotional regulation, Satisfaction with life) ranged from −0.28 to −0.14, and negatively‐worded measures (Sadness, Worry) were 0.21, Table S11]. During the primary school period (−1 ~ 0), students' well‐being remained largely stable (positively‐worded measures ranged from −0.10 to 0.02, negatively‐worded measures were 0.01, Table S11). Well‐being experienced adverse changes during the first‐year post‐transition (0 ~ +1: positively‐worded measures ranged from −0.22 to −0.10; negatively‐worded measures ranged from 0.06 to 0.08), which persisted into the second‐year post‐transition (+1 ~ +2: positively‐worded measures ranged from −0.22 to −0.07; negatively‐worded measures ranged from 0.02 to 0.05). The largest decline during the first 2 years after the transition was shown in cognitive engagement (0 ~ +2: −0.44) and perseverance (0 ~ +2: −0.31).

### Well‐being changes across age cohorts during the school transition

Both cohorts exhibited parallel deteriorations in well‐being during the transition period (0 ~ +1: positively‐worded measures: Old: −0.21 to −0.09, Young: −0.23 to −0.10; negatively‐worded measures: Old: 0.05 to 0.07, Young: 0.08 to 0.09, Table S12, Figure 3). Younger cohort showed larger adverse changes in well‐being during the second‐year post‐transition (+1 ~ +2: positively‐worded measures: Old: −0.19 to −0.04, Young: −0.25 to −0.09; negatively‐worded measures: Old: −0.00 to 0.03, Young: 0.03 to 0.07).

### Well‐being changes across sex during the school transition

Prior to the transition, well‐being scores were comparable between males and females (−1 ~ 0: positively‐worded measures: Female: −0.12 to 0.01, Male: −0.07 to 0.03; negatively‐worded measures: Female: 0.05, Male: −0.03 to −0.02, Table S13, Figure 4). Well‐being showed adverse changes for both sexes in the first 2 years after the transition, with these changes being more pronounced among females (0 ~ +2: positively‐worded measures: Female: −0.52 to −0.26, Male: −0.36 to −0.09; negatively‐worded measures: Female: 0.21 to 0.25, Male: −0.05 to 0.02). Female well‐being later rebounded or stabilized during years three and four (+2 ~ +4: positively‐worded measures: 0.05 to 0.18; negatively‐worded measures: −0.03 to −0.01).

### Well‐being changes across primary language spoken at home during the school transition

Students who spoke a language other than English at home consistently reported higher levels of well‐being compared to those who spoke English (Figure 5). Both groups experienced adverse changes in well‐being during the transition (0 ~ +1: positively‐worded measures: English: −0.22 to −0.10, LOTE: −0.20 to −0.09; negatively‐worded measures: English: 0.06 to 0.08, LOTE: 0.05 to 0.07, Table S14). During the second‐year post‐transition, English‐speaking students experienced greater deterioration across all well‐being domains (+1 ~ +2: positively‐worded measures: English: −0.23 to −0.09, LOTE: −0.17 to −0.02; negatively‐worded measures: English: 0.02 to 0.06, LOTE: −0.01 to 0.02).

### Well‐being changes across residential region during the school transition

Before the transition (−1 ~ 0), students from remote areas had the highest levels of well‐being. Well‐being showed comparable deteriorations across the three residential regions during the first 2 years post‐transition (0 ~ +2: positively‐worded measures: Major city: −0.44 to −0.16, Inner & outer regional: −0.42 to −0.20, Remote and very remote regional: −0.42 to −0.23; negatively‐worded measures: Major city: 0.06 to 0.11, Inner and outer regional: 0.11 to 0.16, Remote and very remote regional: 0.12 to 0.24, Table S15, Figure 6). Well‐being for students living in major cities and inner and outer regional areas rebounded during the third‐year post‐transition (+2 ~ +3: positively‐worded measures: Major city: 0.00 to 0.09, Inner and outer regional: 0.00 to 0.06; negatively‐worded measures: Major city: −0.04 to −0.03, Inner and outer regional: −0.04 to −0.01). However, well‐being for students living in remote and very remote areas continued to worsen during this period and ultimately became the lowest well‐being among all groups (+2 ~ +3: positively‐worded measures: −0.12 to −0.05; negatively‐worded measures: 0.09 to 0.10).

### Well‐being changes across highest parental education levels during the school transition

Students whose parents had higher levels of education consistently reported higher well‐being (Figure 7). A comparable adverse change in well‐being was observed across all three parental education groups during the first 2 years post‐transition (0 ~ +2: positively‐worded measures: Year 12 or less: −0.49 to −0.19, Diploma: −0.44 to −0.18, Bachelor degree or above: −0.37 to −0.16; negatively‐worded measures: Year 12 or less: 0.02 to 0.09, Diploma: 0.07 to 0.12, Bachelor degree or above: 0.13 to 0.18, Table S16).

## Discussion

### Overview of findings

This study examined longitudinal trajectories of students' well‐being across the primary‐to‐secondary school transition. The results revealed a marked adverse change in well‐being during the transition, which persisted for more than 2 years post‐transition. Younger and older cohorts exhibited parallel changes in well‐being, indicating a causal impact of the transition itself. Female students showed greater reductions in well‐being compared with their male peers. Students from non‐English‐speaking backgrounds and those whose parents possessed higher educational qualifications consistently reported higher overall well‐being.

### Interpretation

Previous studies have consistently reported a decline in students' well‐being during the transition from primary‐to‐secondary school, and our findings align with these results (Jindal‐Snape et al., 2020). A noticeable decline in well‐being was observed during 2019–2020 due to the COVID‐19 pandemic. However, South Australia experienced relatively limited disruption, with school closures lasting only a few weeks, and students' well‐being largely remained stable or recovered during 2020–2021. This pattern suggests that the declines in well‐being observed after the transition are more likely attributable to the transition itself rather than to residual pandemic‐related effects. Our study further found that the negative effects of this transition persisted into the second year, with an impact comparable to that of the first year. This pattern reinforces the understanding that school transition is not a single event but a prolonged adjustment process. One plausible explanation for the second‐year decline is that adaptation challenges may re‐emerge after an initial period of adjustment. Students who appeared to adapt successfully in the first year may face renewed difficulties as academic demands increase, class structures evolve, and peer relationships shift (Jindal‐Snape & Cantali, 2019). Another plausible explanation is the reduction of systemic support in the second year. Schools and teachers often provide intensive assistance during the first year to help students settle in, but this support is typically reduced in the following year when students are perceived as having already adapted (Jindal‐Snape & Cantali, 2019).

Our findings indicate that the adverse changes in well‐being during the transition were comparable between younger and older cohorts, suggesting that the well‐being changes are primarily driven by the transition rather than age progression. Similar conclusions were reported in a study conducted in the 1970s, which employed a natural quasi‐experimental design to compare same‐aged students who either transitioned to a new secondary school or remained in a continuous K–8 school setting (Blyth, Simmons, & Bush, 1978; Simmons, 2017). That study demonstrated that declines in psychosocial adjustment observed at early adolescence could be partly attributable to the disruption associated with school transition (Blyth et al., 1978; Simmons, 2017). In our study, the younger cohort experienced a steeper decline in well‐being in the second‐year post‐transition. This implies that an earlier transition to secondary school (i.e., entering 1 year earlier) may have more pronounced and sustained negative effects on students' well‐being. This may be because younger students are more vulnerable to the negative impacts associated with school transitions (Arens, Yeung, Craven, Watermann, & Hasselhorn, 2013). Another possible explanation is that younger students typically occupy lower positions within the new school hierarchy, which may increase their exposure to pressure or intimidation from older peers (Juvonen, Graham, & Schuster, 2003; Kvalsund, 2000).

Two domains, cognitive engagement and perseverance, showed the largest decline during the 2 years post‐transition. Cognitive engagement refers to students' willingness to put in the effort needed to master skills and succeed academically at school, and has a strong motivational component (Gregory et al., 2022). It includes statements such as, “I work hard on learning,” “When I found something hard, I tried another way,” and “No matter who you are, you can change your intelligence.” Previous studies have reported that the school education environment, as well as relationships with teachers and peers, is associated with students' cognitive engagement (Jindal‐Snape et al., 2020; Li & Xue, 2023). Given that students enter an entirely new school environment after transitioning and must rebuild connections with peers, teachers, and other significant adults during this stressful period, the decline in cognitive engagement scores is not surprising. This phenomenon has been documented in prior research (Jindal‐Snape et al., 2020).

Perseverance domain reflects the capacity to persist with tasks and pursue goals even when difficulties arise (Gregory et al., 2022; Kern et al., 2016). The observed decline in perseverance after the transition may be related to changes in the nature of academic tasks encountered in secondary school. In primary school, students typically face fewer academic burdens that can be completed within shorter time frames. Upon transitioning to secondary school, they face the dual challenge of coping with more complex and time‐consuming coursework while needing to sustain their efforts over extended periods to achieve success. This shift poses difficulties for students in completing academic tasks and attaining satisfactory outcomes (Seifert & Beck Jr, 1984). Consequently, students' responses to statements like “I persist with my schoolwork until completion” or “Once I devise a plan to accomplish something, I adhere to it” may tend to yield lower scores. As high levels of perseverance and cognitive engagement during adolescence are associated with higher graduation rates, marital stability, and enhanced adult well‐being, it is essential to provide support for students during this transitional phase (Eskreis‐Winkler, Duckworth, Shulman, & Beal, 2014; Olsson, McGee, Nada‐Raja, & Williams, 2013).

A greater decline in well‐being among females during the primary–secondary school transition has been reported in previous studies (Bharara, 2020; Jindal‐Snape et al., 2020), findings that are consistent with our results. Prior research shows that girls typically report higher well‐being than boys in primary school but lower well‐being in secondary school (Konu & Lintonen, 2006; Palsdottir, Asgeirsdottir, & Sigfusdottir, 2012). This pattern may reflect sex differences in the social and emotional challenges associated with the transition period. Prior research suggests that girls place greater emphasis on peer relationships, social belonging, and social evaluation, all of which may be disrupted during the move to secondary school (van Rens, Haelermans, Groot, & Maassen van den Brink, 2018). Increased sensitivity to peer comparison, changes in friendship networks, and concerns about social acceptance during early adolescence may therefore contribute to greater declines in well‐being among female students. Consistent with this, girls report higher levels of anxiety and internalizing symptoms during this period, which may heighten vulnerability during the transition (Evans, Borriello, & Field, 2018).

A prior study emphasizes that the experience of the primary‐to‐secondary transition is strongly shaped by what students are transitioning from Kvalsund (2000). Rural school contexts, characterized by smaller size and more integrated peer relations, may therefore provide students with greater social continuity, enabling a smoother adjustment to secondary school when students transition together with familiar peers (Kvalsund, 2000). While we found students from remote areas had better well‐being immediately following the school transition compared with students from major cities and regional areas, by the third post‐transition year, well‐being among remote students dropped markedly, to similar or worse levels than students from major city and regional areas. However, the underlying drivers of this pattern cannot be definitively determined within the scope of the current analyses. One important consideration is the substantial sociodemographic heterogeneity across rural and remote areas in South Australia. These areas encompass highly diverse communities shaped by geography, industry, and population distribution. Some regions, such as those supported by strong agricultural, mining, or tourism sectors, have relatively stable employment and access to services, whereas others experience economic decline. Communities also differ markedly in cultural composition, including areas with substantial Aboriginal populations and strong local ties, as well as communities influenced by seasonal or migrant workforces. These contextual differences translate into variation in household income, educational attainment, housing stability, and access to health, education, and digital infrastructure. As a result, “remote and very remote” should not be interpreted as a uniform category; instead, it encompasses a spectrum of sociodemographic conditions that may differentially shape community resilience, service needs, and well‐being outcomes. Further research aimed at explicitly examining these variations is needed to better understand student well‐being trajectories in rural and remote contexts.

## Strengths and limitations

To our knowledge, this study is the largest population‐level investigation of the primary‐to‐secondary school transition. It extends understanding of school transitions through a unique natural experiment that allowed us to disentangle age effects from transition effects. The comprehensive state‐wide dataset provided robust statistical power and enabled examination of multiple demographic factors.

This study has several limitations. Firstly, the annual WEC was conducted once per school year, which limited temporal precision. The short‐term fluctuations in well‐being immediately before or after the transition may not have been captured. Secondly, this study was based on self‐reported questionnaire data, which may be influenced by transient mood states or social desirability. Thirdly, the data were drawn primarily from government schools, and the study population was from a single Australian state. Thus, caution is warranted when generalizing these findings to non‐government school settings and other contexts. Finally, the primary‐to‐secondary school transition occurred in 2022, only 2 years after the major disruptions caused by the pandemic in 2020. Whether lingering effects of the pandemic may have differentially influenced the transition experiences of younger versus older students entering secondary school in 2022 remains unknown. The pre‐transition period was also affected by the pandemic and therefore may not provide an ideal reference for typical pre‐transition well‐being trajectories.

## Implications

The findings hold several important implications for educational practice and policy. Firstly, the transition from primary‐to‐secondary school has a substantial and sustained impact on students' well‐being. According to life course theory, these declines in well‐being during early life may have lasting effects on students' future well‐being outcomes. This finding warrants greater attention from educators and policymakers and highlights the need for targeted strategies to support students through this critical period and to mitigate potential long‐term impacts on their future well‐being. Given that cognitive engagement and perseverance showed the greatest declines, schools could consider incorporating courses that teach effective learning strategies tailored to secondary education. These programs may help students develop goal‐oriented approaches to cope with increased academic demands. Secondly, since the negative effects on well‐being persist beyond the first year of secondary schooling, transition support should extend for at least 2 years. Thirdly, as female students and those living in remote areas were disproportionately affected, future transition support programs should place greater emphasis on these vulnerable groups.

## Future directions

This study focused solely on the effects of school transition on students' well‐being. Future research may consider examining its impacts on other student outcomes, such as academic performance, absenteeism, and risk behaviors. Furthermore, according to life course theory, the decline in well‐being during the transition period may have lasting implications for students' future well‐being outcomes. Longitudinal follow‐up into adulthood is therefore warranted to fully understand these long‐term effects. Finally, future studies could prioritize the development of support interventions, with particular emphasis on female students and those from remote areas during the transition period. For girls in particular, the transition to secondary school may represent a critical turning point that initiates the well‐being decline in their lives. Exploring what drives this decline during the transition will help shape effective, sex‐sensitive strategies to better support students.

## Conclusion

In this study, we examined longitudinal well‐being trajectories across the primary‐to‐secondary school transition and disentangled transition‐related effects from age‐related developmental change. We found that school transitions negatively affected students' well‐being, with causal impacts that persisted for more than 2 years, and were largely attributable to the transition itself rather than age progression. Cognitive engagement and perseverance emerged as the domains most impacted by the transition, highlighting the challenges students face in establishing new relationships and adapting to increased academic demands. Students from non‐English‐speaking backgrounds and those with higher parental education reported higher well‐being across the study period. Female students and those living in remote or very remote areas experienced the largest well‐being declines during the school transition. These findings underscore the need for targeted, sustained interventions that support students for at least 2 years post‐transition, with particular attention to these vulnerable groups.

## Ethical considerations

The WEC census used a parental opt‐out consent process to optimize participation rates and data representativeness. This project received an ethics exemption from the Human Research Ethics Committee of the University of South Australia on August 29, 2023 (application #202625).

**Key points**

**What's known?**

- The transition from primary‐to‐secondary school is associated with declines in student well‐being. However, few studies have tracked students' well‐being across multiple years following the transition, and none have disentangled age effects from transition effects.

**What's new?**

- School transitions negatively affect students' well‐being, with impacts that persist for more than 2 years and are independent of age‐related developmental changes. Females and students residing in remote areas experienced greater declines in well‐being than their male and urban counterparts.

**What's relevant?**

- These findings highlight the need for transition‐specific support strategies that extend beyond the first year of secondary schooling and prioritize female students and those living in rural or remote areas.

## Supporting information


**Figure S1**. Participants flowchart.
**Table S1**. Definition of well‐being.
**Table S2**. The characteristics of participants across calendar year.
**Table S3**. The characteristics of participants across age cohort.
**Table S4**. Multivariate *F* tests results for eight well‐being domain.
**Table S5**. Marginal mean for eight well‐being sub‐domains over time.
**Table S6**. Marginal mean for eight well‐being sub‐domains over time across age cohort.
**Table S7**. Marginal mean for eight well‐being sub‐domains over time across gender.
**Table S8**. Marginal mean for eight well‐being sub‐domains over time across primary language spoken at home.
**Table S9**. Marginal mean for eight well‐being sub‐domains over time across residential region.
**Table S10**. Marginal mean for eight well‐being sub‐domains over time across highest parental educational level.
**Table S11**. Marginal effects over time.
**Table S12**. Marginal effects over time across age cohort.
**Table S13**. Marginal effects over time across gender.
**Table S14**. Marginal effects over time across main language spoken at home.
**Table S15**. Marginal effects over time across residential region.
**Table S16**. Marginal effects over time across parental highest education.

## Data Availability

The data that support the findings of this study are available upon request from the corresponding author. The data are not publicly available due to privacy or ethical restrictions.
