# Supplementary material for: Well‐being decline during adolescence: school transition as a predominant driver beyond age progression
Source: J Child Psychol Psychiatry. 2026 Apr 21;67(9):1513–24. doi: 10.1111/jcpp.70154 (PMC13450302; doi:10.1111/jcpp.70154)
Supplement: Supplementary file 1 [file JCPP-67-1513-s001.docx]

**Well‑being Decline During Adolescence: School Transition as a Predominant Driver Beyond Age Progression**

**Supporting Information**

Table of Contents

[Fig S1. Participants flowchart	2](#_Toc213672355)

[Table S1 Definition of wellbeing	3](#_Toc213672356)

[Table S2 The characteristics of participants across calendar year	4](#_Toc213672357)

[Table S3 The characteristics of participants across age cohort	5](#_Toc213672358)

[Table S4. Multivariate F tests results for eight wellbeing domains	6](#_Toc213672359)

[Table S5. Marginal mean for eight wellbeing sub-domains over time	7](#_Toc213672360)

[Table S6. Marginal mean for eight wellbeing sub-domains over time across age cohort	8](#_Toc213672361)

[Table S7. Marginal mean for eight wellbeing sub-domains over time across gender	9](#_Toc213672362)

[Table S8. Marginal mean for eight wellbeing sub-domains over time across primary language spoken at home	10](#_Toc213672363)

[Table S9. Marginal mean for eight wellbeing sub-domains over time across residential region	11](#_Toc213672364)

[Table S10. Marginal mean for eight wellbeing sub-domains over time across highest parental educational level	12](#_Toc213672365)

[Table S11. Marginal effects over time	13](#_Toc213672366)

[Table S12. Marginal effects over time across age cohort	14](#_Toc213672367)

[Table S13. Marginal effects over time across gender	15](#_Toc213672368)

[Table S14. Marginal effects over time across main language spoken at home	16](#_Toc213672369)

[Table S15. Marginal effects over time across residential region	17](#_Toc213672370)

[Table S16. Marginal effects over time across parental highest education	18](#_Toc213672371)

Fig S1. Participants flowchart

Table S1. Definition of wellbeing

| **Wellbeing item** | **Definition** |
| --- | --- |
| Cognitive engagement | Persistence with classroom tasks, generating ideas and attitudes related, holding a growth mindset |
| Happiness | General feeling of happiness, cheer and contentment with life |
| Optimism | Having a mindset of positive expectations about the future |
| Perseverance | Having the tenacity, stick with things and pursue goals, despite challenges that arise |
| Emotion regulation | Having the ability, manage the experience of positive and negative feelings |
| Satisfaction with life | How content or satisfied children are with their lives |
| Sadness | How frequently young people feel unhappy or upset |
| Worry | How often young people worry about different aspects of life |

Table S2. The characteristics of participants across calendar year

| **Characteristics** | **Levels** | **Two years before transition (-2)** | **One year before transition (-1)** | **Final year of primary school (0)** | **First year post transition (+1)** | **Second year post transition (+2)** | **Third year post transition (+3)** | **Fourth year post transition (+4)** |
| --- | --- | --- | --- | --- | --- | --- | --- | --- |
| **Calendar year** |  | 2019 (N = 14820) | 2020 (N=13761) | 2021 (N=17761) | 2022 (N=16101) | 2023 (N=15496) | 2024 (N=14183) | 2025 (N=12678) |
| **Age (y)** | Mean ± SD | 9.7 ± 0.6 | 10.7 ± 0.6 | 11.7 ± 0.6 | 12.7 ± 0.6 | 13.7 ± 0.6 | 14.7 ± 0.6 | 15.3 ± 0.6 |
| **Gender, n (%)** | Female | 7148 (48.2%) | 6638 (48.2%) | 8534 (48%) | 7812 (48.5%) | 7368 (47.5%) | 6699 (47.2%) | 6049 (47.7%) |
|  | Male | 7672 (51.8%) | 7123 (51.8%) | 9227 (52%) | 8289 (51.5%) | 8128 (52.5%) | 7484 (52.8%) | 6629 (52.3%) |
| **Primary language spoken at home, n (%)** | English | 11593 (78.2%) | 10741 (78.1%) | 13657 (76.9%) | 12256 (76.1%) | 11921 (76.9%) | 10973 (77.4%) | 9831 (77.5%) |
|  | Not English | 3227 (21.8%) | 3020 (21.9%) | 4104 (23.1%) | 3845 (23.9%) | 3575 (23.1%) | 3210 (22.6%) | 2847 (22.5%) |
| **Residential region, n (%)** | Major city | 10471 (70.7%) | 9506 (69.1%) | 12639 (71.2%) | 11468 (71.2%) | 10959 (70.7%) | 10000 (70.5%) | 8938 (70.5%) |
|  | Inner & outer regional | 3868 (26.1%) | 3603 (26.2%) | 4463 (25.1%) | 4034 (25.1%) | 3961 (25.6%) | 3673 (25.9%) | 3321 (26.2%) |
|  | Remote & very remote regional | 481 (3.2%) | 652 (4.7%) | 659 (3.7%) | 599 (3.7%) | 576 (3.7%) | 510 (3.6%) | 419 (3.3%) |
| **Highest parental education level, n (%)** | Year 12 or less | 3060 (20.6%) | 2715 (19.7%) | 3541 (19.9%) | 2752 (17.1%) | 2498 (16.1%) | 2222 (15.7%) | 2075 (16.4%) |
|  | Diploma | 7036 (47.5%) | 6682 (48.6%) | 8426 (47.4%) | 7777 (48.3%) | 7527 (48.6%) | 6890 (48.6%) | 6040 (47.6%) |
|  | Bachelor degree or above | 4724 (31.9%) | 4364 (31.7%) | 5794 (32.6%) | 5572 (34.6%) | 5471 (35.3%) | 5071 (35.8%) | 4563 (36%) |
| **Change in physical school environment during the transition, n (%)** | No | 1285 (8.7%) | 1427 (10.4%) | 1876 (10.6%) | 1733 (10.8%) | 1744 (11.3%) | 1524 (10.7%) | 1271 (10%) |
|  | Yes | 13535 (91.3%) | 12334 (89.6%) | 15885 (89.4%) | 14368 (89.2%) | 13752 (88.7%) | 12659 (89.3%) | 11407 (90%) |
| **Age cohort, n (%)** | Old | 7559 (51%) | 6776 (49.2%) | 8867 (49.9%) | 8119 (50.4%) | 7617 (49.2%) | 6888 (48.6%) | 5997 (47.3%) |
|  | Young | 7261 (49%) | 6985 (50.8%) | 8894 (50.1%) | 7982 (49.6%) | 7879 (50.8%) | 7295 (51.4%) | 6681 (52.7%) |
| **Grade, n (%)** | 4 | 7261 (49%) | 0 (0%) | 0 (0%) | 0 (0%) | 0 (0%) | 0 (0%) | 0 (0%) |
|  | 5 | 7559 (51%) | 6985 (50.8%) | 0 (0%) | 0 (0%) | 0 (0%) | 0 (0%) | 0 (0%) |
|  | 6 | 0 (0%) | 6776 (49.2%) | 8894 (50.1%) | 0 (0%) | 0 (0%) | 0 (0%) | 0 (0%) |
|  | 7 | 0 (0%) | 0 (0%) | 8867 (49.9%) | 7982 (49.6%) | 0 (0%) | 0 (0%) | 0 (0%) |
|  | 8 | 0 (0%) | 0 (0%) | 0 (0%) | 8119 (50.4%) | 7879 (50.8%) | 0 (0%) | 0 (0%) |
|  | 9 | 0 (0%) | 0 (0%) | 0 (0%) | 0 (0%) | 7617 (49.2%) | 7295 (51.4%) | 0 (0%) |
|  | 10 | 0 (0%) | 0 (0%) | 0 (0%) | 0 (0%) | 0 (0%) | 6888 (48.6%) | 6681 (52.7%) |
|  | 11 | 0 (0%) | 0 (0%) | 0 (0%) | 0 (0%) | 0 (0%) | 0 (0%) | 5997 (47.3%) |
| **Wellbeing domain** | | | | | | | | |
| **Cognitive engagement** | Mean ± SD | 4.0 ± 0.8 | 3.8 ± 0.8 | 3.8 ± 0.8 | 3.6 ± 0.8 | 3.4 ± 0.9 | 3.4 ± 0.8 | 3.4 ± 0.8 |
| **Happiness** | Mean ± SD | 4.0 ± 0.7 | 3.8 ± 0.8 | 3.8 ± 0.8 | 3.7 ± 0.7 | 3.6 ± 0.8 | 3.7 ± 0.7 | 3.6 ± 0.8 |
| **Optimism** | Mean ± SD | 3.9 ± 0.9 | 3.7 ± 0.9 | 3.6 ± 0.9 | 3.5 ± 0.9 | 3.4 ± 0.9 | 3.5 ± 0.8 | 3.5 ± 0.8 |
| **Perseverance** | Mean ± SD | 3.8 ± 0.7 | 3.7 ± 0.7 | 3.7 ± 0.7 | 3.5 ± 0.7 | 3.4 ± 0.8 | 3.4 ± 0.8 | 3.4 ± 0.8 |
| **Emotional regulation** | Mean ± SD | 3.6 ± 0.9 | 3.4 ± 0.9 | 3.3 ± 0.9 | 3.2 ± 0.9 | 3.1 ± 0.9 | 3.2 ± 0.9 | 3.2 ± 0.9 |
| **Satisfaction with life** | Mean ± SD | 3.8 ± 0.9 | 3.6 ± 0.9 | 3.6 ± 0.9 | 3.5 ± 0.9 | 3.4 ± 0.9 | 3.4 ± 0.9 | 3.4 ± 0.9 |
| **Sadness** | Mean ± SD | 2.6 ± 1.0 | 2.8 ± 1.0 | 2.8 ± 1.0 | 2.9 ± 0.9 | 3.0 ± 1.0 | 2.9 ± 1.0 | 2.9 ± 1.0 |
| **Worry** | Mean ± SD | 2.9 ± 1.1 | 3.1 ± 1.0 | 3.1 ± 1.0 | 3.2 ± 1.0 | 3.2 ± 1.0 | 3.1 ± 1.0 | 3.2 ± 1.0 |

Table S3. The characteristics of participants across age cohort

| **Characteristics** | **Levels** | **Old (N=51823)** | **Young (N=52977)** |
| --- | --- | --- | --- |
| **Age (y)** | Mean ± SD | 13.0 ± 1.9 | 12.1 ± 1.9 |
| **Gender, n (%)** | Female | 24984 (48.2%) | 25264 (47.7%) |
|  | Male | 26839 (51.8%) | 27713 (52.3%) |
| **Primary language spoken at home, n (%)** | English | 40154 (77.5%) | 40818 (77%) |
|  | Not English | 11669 (22.5%) | 12159 (23%) |
| **Residential region, n (%)** | Major city | 36505 (70.4%) | 37476 (70.7%) |
|  | Inner & outer regional | 13290 (25.6%) | 13633 (25.7%) |
|  | Remote & very remote regional | 2028 (3.9%) | 1868 (3.5%) |
| **Highest parental education level, n (%)** | Year 12 or less | 9395 (18.1%) | 9468 (17.9%) |
|  | Diploma | 25182 (48.6%) | 25196 (47.6%) |
|  | Bachelor degree or above | 17246 (33.3%) | 18313 (34.6%) |
| **Change in physical school environment during the transition, n (%)** | No | 5277 (10.2%) | 5583 (10.5%) |
|  | Yes | 46546 (89.8%) | 47394 (89.5%) |
| **Calendar year, n (%)** | 2019 | 7559 (14.6%) | 7261 (13.7%) |
|  | 2020 | 6776 (13.1%) | 6985 (13.2%) |
|  | 2021 | 8867 (17.1%) | 8894 (16.8%) |
|  | 2022 | 8119 (15.7%) | 7982 (15.1%) |
|  | 2023 | 7617 (14.7%) | 7879 (14.9%) |
|  | 2024 | 6888 (13.3%) | 7295 (13.8%) |
|  | 2025 | 5997 (11.6%) | 6681 (12.6%) |
| **Grade, n (%)** | 4 | 0 (0%) | 7261 (13.7%) |
|  | 5 | 7559 (14.6%) | 6985 (13.2%) |
|  | 6 | 6776 (13.1%) | 8894 (16.8%) |
|  | 7 | 8867 (17.1%) | 7982 (15.1%) |
|  | 8 | 8119 (15.7%) | 7879 (14.9%) |
|  | 9 | 7617 (14.7%) | 7295 (13.8%) |
|  | 10 | 6888 (13.3%) | 6681 (12.6%) |
|  | 11 | 5997 (11.6%) | 0 (0%) |
| **Wellbeing domain** | | | |
| **Cognitive engagement** | Mean ± SD | 3.6 ± 0.8 | 3.7 ± 0.8 |
| **Happiness** | Mean ± SD | 3.7 ± 0.8 | 3.7 ± 0.8 |
| **Optimism** | Mean ± SD | 3.6 ± 0.9 | 3.6 ± 0.9 |
| **Perseverance** | Mean ± SD | 3.6 ± 0.8 | 3.6 ± 0.8 |
| **Emotional regulation** | Mean ± SD | 3.3 ± 0.9 | 3.3 ± 0.9 |
| **Satisfaction with life** | Mean ± SD | 3.5 ± 0.9 | 3.6 ± 0.9 |
| **Sadness** | Mean ± SD | 2.9 ± 1.0 | 2.8 ± 1.0 |
| **Worry** | Mean ± SD | 3.1 ± 1.0 | 3.1 ± 1.0 |

Table S4. Multivariate F tests results for eight wellbeing domains

|  | **Cognitive engagement**  **(N = 100088)** | | **Happiness**  **(N = 103364)** | | **Optimism**  **(N = 102892)** | | **Perseverance**  **(N = 103099)** | | **Emotional regulation**  **(N = 102414)** | | **Satisfaction with life**  **(N = 102796)** | | **Sadness**  **(N = 102475)** | | **Worry**  **(N = 102338)** | |
| --- | --- | --- | --- | --- | --- | --- | --- | --- | --- | --- | --- | --- | --- | --- | --- | --- |
|  | χ² | P value | Χ² | P value | Χ² | P value | Χ² | P value | Χ² | P value | Χ² | P value | Χ² | P value | Χ² | P value |
| Calendar Year | 4351.91 | <0.001 | 1939.66 | <0.001 | 2448.51 | <0.001 | 2577.13 | <0.001 | 2871.70 | <0.001 | 2377.19 | <0.001 | 1120.02 | <0.001 | 834.88 | <0.001 |
| Gender | 20.52 | <0.001 | 279.30 | <0.001 | 419.98 | <0.001 | 2.60 | 0.107 | 337.17 | <0.001 | 607.15 | <0.001 | 1338.86 | <0.001 | 1757.28 | <0.001 |
| Change in physical school environment during the transition | 0.02 | 0.902 | 4.48 | 0.034 | 7.60 | 0.006 | 0.72 | 0.398 | 3.23 | 0.072 | 13.38 | <0.001 | 8.06 | 0.005 | 7.06 | 0.008 |
| Residential region | 0.64 | 0.726 | 2.45 | 0.293 | 5.53 | 0.063 | 3.34 | 0.188 | 10.25 | 0.006 | 3.76 | 0.152 | 0.43 | 0.806 | 1.63 | 0.443 |
| Primary language spoken at home | 160.22 | <0.001 | 53.20 | <0.001 | 119.69 | <0.001 | 109.81 | <0.001 | 235.17 | <0.001 | 34.34 | <0.001 | 85.45 | <0.001 | 36.06 | <0.001 |
| Highest parental education level | 427.72 | <0.001 | 142.90 | <0.001 | 200.63 | <0.001 | 418.40 | <0.001 | 42.43 | <0.001 | 126.67 | <0.001 | 332.66 | <0.001 | 177.19 | <0.001 |

Table S5. Marginal mean for eight wellbeing sub-domains over time

| **Years since transition** | **Cognitive engagement**  **(N = 100088)** | **Happiness**  **(N = 103364)** | **Optimism**  **(N = 102892)** | **Perseverance**  **(N = 103099)** | **Emotional regulation**  **(N = 102414)** | **Satisfaction with life**  **(N = 102796)** | **Sadness**  **(N = 102475)** | **Worry**  **(N = 102338)** |
| --- | --- | --- | --- | --- | --- | --- | --- | --- |
| Two years before transition (-2) | 4 (3.98, 4.02) | 3.99 (3.97, 4) | 3.91 (3.89, 3.92) | 3.83 (3.82, 3.85) | 3.67 (3.65, 3.69) | 3.85 (3.83, 3.87) | 2.61 (2.59, 2.63) | 2.89 (2.87, 2.91) |
| One year before transition (-1) | 3.81 (3.79, 3.83) | 3.79 (3.78, 3.81) | 3.66 (3.64, 3.68) | 3.69 (3.68, 3.71) | 3.39 (3.37, 3.41) | 3.66 (3.64, 3.67) | 2.82 (2.8, 2.84) | 3.1 (3.08, 3.12) |
| Final year of primary school (0) | 3.83 (3.82, 3.85) | 3.78 (3.77, 3.79) | 3.62 (3.61, 3.64) | 3.7 (3.69, 3.72) | 3.3 (3.28, 3.31) | 3.62 (3.6, 3.63) | 2.83 (2.82, 2.85) | 3.11 (3.09, 3.12) |
| First year post transition (+1) | 3.4 (3.38, 3.42) | 3.6 (3.59, 3.62) | 3.42 (3.4, 3.44) | 3.39 (3.37, 3.41) | 3.1 (3.08, 3.12) | 3.38 (3.36, 3.4) | 2.96 (2.94, 2.99) | 3.18 (3.16, 3.21) |
| Second year post transition (+2) | 3.61 (3.6, 3.63) | 3.68 (3.66, 3.7) | 3.52 (3.5, 3.54) | 3.54 (3.52, 3.56) | 3.17 (3.15, 3.19) | 3.5 (3.48, 3.52) | 2.91 (2.89, 2.94) | 3.17 (3.15, 3.19) |
| Third year post transition (+3) | 3.4 (3.38, 3.42) | 3.64 (3.62, 3.66) | 3.47 (3.45, 3.49) | 3.41 (3.39, 3.43) | 3.18 (3.15, 3.2) | 3.4 (3.38, 3.42) | 2.93 (2.91, 2.95) | 3.16 (3.14, 3.18) |
| Fourth year post transition (+4) | 3.41 (3.39, 3.43) | 3.63 (3.61, 3.65) | 3.47 (3.45, 3.49) | 3.43 (3.41, 3.45) | 3.22 (3.2, 3.24) | 3.39 (3.37, 3.41) | 2.94 (2.91, 2.96) | 3.18 (3.15, 3.2) |

Table S6. Marginal mean for eight wellbeing sub-domains over time across age cohort

| **Group** | **Years since transition** | **Cognitive engagement**  **(N = 100088)** | **Happiness**  **(N = 103364)** | **Optimism**  **(N = 102892)** | **Perseverance**  **(N = 103099)** | **Emotional regulation**  **(N = 102414)** | **Satisfaction with life**  **(N = 102796)** | **Sadness**  **(N = 102475)** | **Worry**  **(N = 102338)** |
| --- | --- | --- | --- | --- | --- | --- | --- | --- | --- |
| Young | Two years before transition (-2) | 4.01 (3.99, 4.04) | 4 (3.98, 4.02) | 3.93 (3.91, 3.95) | 3.85 (3.83, 3.86) | 3.73 (3.71, 3.75) | 3.86 (3.84, 3.88) | 2.61 (2.59, 2.63) | 2.91 (2.88, 2.93) |
|  | One year before transition (-1) | 3.86 (3.84, 3.88) | 3.82 (3.8, 3.83) | 3.69 (3.66, 3.71) | 3.72 (3.7, 3.74) | 3.45 (3.43, 3.47) | 3.7 (3.67, 3.72) | 2.8 (2.77, 2.82) | 3.08 (3.05, 3.1) |
|  | Final year of primary school (0) | 3.88 (3.86, 3.9) | 3.81 (3.79, 3.83) | 3.66 (3.64, 3.68) | 3.73 (3.72, 3.75) | 3.35 (3.33, 3.37) | 3.67 (3.65, 3.69) | 2.8 (2.78, 2.83) | 3.09 (3.06, 3.11) |
|  | First year post transition (+1) | 3.41 (3.39, 3.43) | 3.62 (3.6, 3.64) | 3.43 (3.41, 3.46) | 3.39 (3.37, 3.41) | 3.1 (3.08, 3.13) | 3.41 (3.39, 3.44) | 2.96 (2.94, 2.99) | 3.2 (3.17, 3.22) |
|  | Second year post transition (+2) | 3.66 (3.63, 3.68) | 3.71 (3.69, 3.73) | 3.54 (3.52, 3.57) | 3.57 (3.55, 3.59) | 3.2 (3.18, 3.23) | 3.55 (3.53, 3.57) | 2.9 (2.87, 2.92) | 3.16 (3.14, 3.19) |
|  | Third year post transition (+3) | 3.39 (3.37, 3.42) | 3.64 (3.62, 3.66) | 3.46 (3.43, 3.48) | 3.39 (3.37, 3.41) | 3.15 (3.12, 3.17) | 3.41 (3.38, 3.43) | 2.93 (2.91, 2.96) | 3.14 (3.11, 3.17) |
|  | Fourth year post transition (+4) | 3.4 (3.38, 3.43) | 3.66 (3.63, 3.68) | 3.49 (3.46, 3.51) | 3.43 (3.41, 3.45) | 3.23 (3.21, 3.26) | 3.41 (3.39, 3.44) | 2.93 (2.9, 2.95) | 3.16 (3.13, 3.19) |
| Old | Two years before transition (-2) | 3.99 (3.97, 4.01) | 3.98 (3.96, 4) | 3.88 (3.86, 3.9) | 3.82 (3.8, 3.84) | 3.61 (3.59, 3.63) | 3.84 (3.82, 3.86) | 2.61 (2.59, 2.64) | 2.88 (2.85, 2.9) |
|  | One year before transition (-1) | 3.76 (3.74, 3.79) | 3.77 (3.75, 3.79) | 3.64 (3.61, 3.66) | 3.67 (3.65, 3.69) | 3.34 (3.31, 3.36) | 3.62 (3.6, 3.64) | 2.84 (2.82, 2.87) | 3.12 (3.1, 3.15) |
|  | Final year of primary school (0) | 3.78 (3.77, 3.8) | 3.75 (3.73, 3.77) | 3.58 (3.56, 3.6) | 3.67 (3.65, 3.69) | 3.24 (3.22, 3.27) | 3.57 (3.55, 3.59) | 2.86 (2.84, 2.88) | 3.13 (3.1, 3.15) |
|  | First year post transition (+1) | 3.38 (3.36, 3.41) | 3.59 (3.56, 3.61) | 3.4 (3.38, 3.43) | 3.39 (3.37, 3.42) | 3.1 (3.07, 3.12) | 3.34 (3.32, 3.36) | 2.96 (2.94, 2.99) | 3.17 (3.14, 3.2) |
|  | Second year post transition (+2) | 3.57 (3.55, 3.59) | 3.65 (3.63, 3.67) | 3.49 (3.47, 3.51) | 3.51 (3.49, 3.54) | 3.14 (3.11, 3.16) | 3.45 (3.43, 3.48) | 2.93 (2.91, 2.96) | 3.17 (3.15, 3.2) |
|  | Third year post transition (+3) | 3.4 (3.38, 3.43) | 3.64 (3.62, 3.66) | 3.48 (3.45, 3.5) | 3.43 (3.41, 3.45) | 3.21 (3.18, 3.23) | 3.4 (3.37, 3.42) | 2.93 (2.9, 2.96) | 3.18 (3.15, 3.21) |
|  | Fourth year post transition (+4) | 3.42 (3.39, 3.44) | 3.6 (3.58, 3.63) | 3.46 (3.44, 3.49) | 3.43 (3.41, 3.45) | 3.21 (3.18, 3.23) | 3.37 (3.34, 3.39) | 2.94 (2.92, 2.97) | 3.19 (3.16, 3.22) |

Table S7. Marginal mean for eight wellbeing sub-domains over time across gender

| **Gender** | **Years since transition** | **Cognitive engagement**  **(N = 100088)** | **Happiness**  **(N = 103364)** | **Optimism**  **(N = 102892)** | **Perseverance**  **(N = 103099)** | **Emotional regulation**  **(N = 102414)** | **Satisfaction with life**  **(N = 102796)** | **Sadness**  **(N = 102475)** | **Worry**  **(N = 102338)** |
| --- | --- | --- | --- | --- | --- | --- | --- | --- | --- |
| Male | Two years before transition (-2) | 3.91 (3.89, 3.93) | 3.93 (3.92, 3.95) | 3.86 (3.84, 3.88) | 3.74 (3.72, 3.75) | 3.63 (3.61, 3.65) | 3.84 (3.82, 3.86) | 2.6 (2.57, 2.62) | 2.86 (2.84, 2.89) |
|  | One year before transition (-1) | 3.76 (3.74, 3.78) | 3.81 (3.79, 3.83) | 3.7 (3.68, 3.73) | 3.64 (3.63, 3.66) | 3.42 (3.4, 3.44) | 3.72 (3.7, 3.74) | 2.72 (2.7, 2.75) | 2.97 (2.95, 3) |
|  | Final year of primary school (0) | 3.79 (3.77, 3.81) | 3.82 (3.81, 3.84) | 3.69 (3.67, 3.71) | 3.67 (3.65, 3.69) | 3.35 (3.33, 3.37) | 3.7 (3.69, 3.72) | 2.7 (2.68, 2.72) | 2.94 (2.92, 2.96) |
|  | First year post transition (+1) | 3.44 (3.42, 3.46) | 3.73 (3.71, 3.75) | 3.58 (3.55, 3.6) | 3.47 (3.45, 3.49) | 3.26 (3.23, 3.28) | 3.56 (3.53, 3.58) | 2.72 (2.69, 2.74) | 2.89 (2.87, 2.92) |
|  | Second year post transition (+2) | 3.62 (3.59, 3.64) | 3.77 (3.75, 3.79) | 3.62 (3.6, 3.65) | 3.57 (3.54, 3.59) | 3.28 (3.26, 3.3) | 3.64 (3.61, 3.66) | 2.73 (2.7, 2.75) | 2.94 (2.92, 2.97) |
|  | Third year post transition (+3) | 3.42 (3.4, 3.44) | 3.74 (3.72, 3.77) | 3.61 (3.58, 3.63) | 3.48 (3.46, 3.5) | 3.29 (3.27, 3.32) | 3.56 (3.54, 3.58) | 2.69 (2.66, 2.71) | 2.86 (2.84, 2.89) |
|  | Fourth year post transition (+4) | 3.41 (3.39, 3.44) | 3.72 (3.7, 3.74) | 3.6 (3.57, 3.62) | 3.48 (3.46, 3.5) | 3.32 (3.3, 3.35) | 3.53 (3.5, 3.55) | 2.69 (2.67, 2.72) | 2.88 (2.86, 2.91) |
| Female | Two years before transition (-2) | 4.09 (4.07, 4.12) | 4.04 (4.02, 4.06) | 3.96 (3.93, 3.98) | 3.93 (3.92, 3.95) | 3.71 (3.69, 3.73) | 3.86 (3.84, 3.88) | 2.63 (2.61, 2.66) | 2.93 (2.9, 2.95) |
|  | One year before transition (-1) | 3.86 (3.84, 3.88) | 3.77 (3.75, 3.79) | 3.61 (3.59, 3.63) | 3.74 (3.72, 3.76) | 3.36 (3.33, 3.38) | 3.58 (3.56, 3.61) | 2.93 (2.9, 2.95) | 3.24 (3.21, 3.27) |
|  | Final year of primary school (0) | 3.88 (3.86, 3.9) | 3.73 (3.72, 3.75) | 3.55 (3.53, 3.57) | 3.74 (3.72, 3.75) | 3.23 (3.21, 3.26) | 3.52 (3.5, 3.54) | 2.98 (2.96, 3) | 3.29 (3.26, 3.31) |
|  | First year post transition (+1) | 3.35 (3.33, 3.38) | 3.47 (3.45, 3.49) | 3.25 (3.22, 3.27) | 3.31 (3.28, 3.33) | 2.93 (2.9, 2.95) | 3.18 (3.16, 3.2) | 3.23 (3.2, 3.25) | 3.5 (3.47, 3.52) |
|  | Second year post transition (+2) | 3.62 (3.59, 3.64) | 3.58 (3.56, 3.6) | 3.41 (3.38, 3.43) | 3.52 (3.5, 3.54) | 3.06 (3.03, 3.08) | 3.36 (3.34, 3.38) | 3.11 (3.09, 3.14) | 3.41 (3.38, 3.44) |
|  | Third year post transition (+3) | 3.38 (3.35, 3.4) | 3.53 (3.5, 3.55) | 3.31 (3.29, 3.34) | 3.33 (3.31, 3.35) | 3.05 (3.02, 3.07) | 3.23 (3.21, 3.25) | 3.2 (3.17, 3.22) | 3.48 (3.45, 3.5) |
|  | Fourth year post transition (+4) | 3.4 (3.38, 3.43) | 3.53 (3.51, 3.56) | 3.34 (3.31, 3.36) | 3.38 (3.36, 3.4) | 3.11 (3.08, 3.14) | 3.25 (3.22, 3.27) | 3.19 (3.17, 3.22) | 3.49 (3.46, 3.52) |

Table S8. Marginal mean for eight wellbeing sub-domains over time across primary language spoken at home

| **Primary language spoken at home** | **Years since transition** | **Cognitive engagement**  **(N = 100088)** | **Happiness**  **(N = 103364)** | **Optimism**  **(N = 102892)** | **Perseverance**  **(N = 103099)** | **Emotional regulation**  **(N = 102414)** | **Satisfaction with life**  **(N = 102796)** | **Sadness**  **(N = 102475)** | **Worry**  **(N = 102338)** |
| --- | --- | --- | --- | --- | --- | --- | --- | --- | --- |
| English | Two years before transition (-2) | 3.99 (3.98, 4.01) | 3.98 (3.97, 4) | 3.89 (3.87, 3.91) | 3.83 (3.81, 3.84) | 3.65 (3.63, 3.67) | 3.84 (3.82, 3.86) | 2.63 (2.61, 2.65) | 2.9 (2.88, 2.92) |
|  | One year before transition (-1) | 3.78 (3.77, 3.8) | 3.77 (3.76, 3.79) | 3.64 (3.62, 3.66) | 3.67 (3.66, 3.69) | 3.36 (3.34, 3.38) | 3.64 (3.63, 3.66) | 2.85 (2.83, 2.87) | 3.11 (3.09, 3.13) |
|  | Final year of primary school (0) | 3.81 (3.79, 3.82) | 3.77 (3.76, 3.79) | 3.6 (3.58, 3.62) | 3.68 (3.67, 3.7) | 3.26 (3.24, 3.28) | 3.61 (3.59, 3.63) | 2.85 (2.83, 2.87) | 3.12 (3.1, 3.14) |
|  | First year post transition (+1) | 3.36 (3.34, 3.38) | 3.58 (3.56, 3.6) | 3.39 (3.36, 3.41) | 3.36 (3.34, 3.38) | 3.05 (3.03, 3.08) | 3.36 (3.34, 3.38) | 2.99 (2.97, 3.01) | 3.2 (3.18, 3.23) |
|  | Second year post transition (+2) | 3.59 (3.57, 3.61) | 3.67 (3.65, 3.69) | 3.5 (3.48, 3.52) | 3.52 (3.51, 3.54) | 3.14 (3.12, 3.16) | 3.5 (3.48, 3.52) | 2.93 (2.91, 2.96) | 3.18 (3.16, 3.21) |
|  | Third year post transition (+3) | 3.35 (3.33, 3.38) | 3.62 (3.6, 3.64) | 3.43 (3.41, 3.45) | 3.38 (3.36, 3.4) | 3.13 (3.1, 3.15) | 3.38 (3.36, 3.4) | 2.96 (2.94, 2.99) | 3.18 (3.16, 3.21) |
|  | Fourth year post transition (+4) | 3.37 (3.35, 3.4) | 3.61 (3.59, 3.63) | 3.45 (3.42, 3.47) | 3.41 (3.39, 3.43) | 3.18 (3.15, 3.2) | 3.37 (3.35, 3.39) | 2.96 (2.94, 2.99) | 3.19 (3.17, 3.22) |
| LOTE | Two years before transition (-2) | 4 (3.97, 4.03) | 3.99 (3.97, 4.02) | 3.96 (3.93, 3.99) | 3.84 (3.81, 3.86) | 3.72 (3.69, 3.75) | 3.88 (3.85, 3.91) | 2.58 (2.55, 2.61) | 2.87 (2.84, 2.91) |
|  | One year before transition (-1) | 3.89 (3.86, 3.91) | 3.84 (3.81, 3.87) | 3.74 (3.7, 3.77) | 3.74 (3.72, 3.77) | 3.5 (3.46, 3.53) | 3.69 (3.66, 3.73) | 2.74 (2.7, 2.77) | 3.07 (3.03, 3.11) |
|  | Final year of primary school (0) | 3.9 (3.88, 3.93) | 3.8 (3.78, 3.83) | 3.69 (3.66, 3.72) | 3.76 (3.73, 3.78) | 3.41 (3.38, 3.44) | 3.65 (3.62, 3.68) | 2.77 (2.74, 2.8) | 3.06 (3.03, 3.1) |
|  | First year post transition (+1) | 3.54 (3.51, 3.57) | 3.7 (3.67, 3.72) | 3.53 (3.5, 3.57) | 3.5 (3.47, 3.53) | 3.26 (3.23, 3.3) | 3.45 (3.42, 3.48) | 2.86 (2.83, 2.9) | 3.11 (3.07, 3.14) |
|  | Second year post transition (+2) | 3.7 (3.67, 3.73) | 3.72 (3.69, 3.74) | 3.58 (3.55, 3.61) | 3.6 (3.58, 3.63) | 3.28 (3.25, 3.32) | 3.52 (3.49, 3.55) | 2.84 (2.81, 2.88) | 3.11 (3.08, 3.15) |
|  | Third year post transition (+3) | 3.56 (3.53, 3.59) | 3.72 (3.69, 3.75) | 3.59 (3.56, 3.62) | 3.53 (3.5, 3.56) | 3.35 (3.32, 3.39) | 3.48 (3.45, 3.52) | 2.82 (2.79, 2.86) | 3.07 (3.04, 3.11) |
|  | Fourth year post transition (+4) | 3.53 (3.5, 3.57) | 3.7 (3.67, 3.73) | 3.57 (3.54, 3.61) | 3.52 (3.49, 3.55) | 3.37 (3.34, 3.41) | 3.47 (3.43, 3.5) | 2.84 (2.8, 2.87) | 3.11 (3.07, 3.15) |

Table S9. Marginal mean for eight wellbeing sub-domains over time across residential region

| **Residential region** | **Years since transition** | **Cognitive engagement**  **(N = 100088)** | **Happiness**  **(N = 103364)** | **Optimism**  **(N = 102892)** | **Perseverance**  **(N = 103099)** | **Emotional regulation**  **(N = 102414)** | **Satisfaction with life**  **(N = 102796)** | **Sadness**  **(N = 102475)** | **Worry**  **(N = 102338)** |
| --- | --- | --- | --- | --- | --- | --- | --- | --- | --- |
| Major city | Two years before transition (-2) | 4 (3.98, 4.02) | 3.98 (3.96, 4) | 3.9 (3.88, 3.92) | 3.84 (3.82, 3.86) | 3.65 (3.62, 3.67) | 3.84 (3.83, 3.86) | 2.61 (2.58, 2.63) | 2.89 (2.86, 2.91) |
|  | One year before transition (-1) | 3.81 (3.79, 3.83) | 3.78 (3.76, 3.8) | 3.65 (3.63, 3.67) | 3.7 (3.68, 3.72) | 3.37 (3.35, 3.39) | 3.65 (3.63, 3.67) | 2.83 (2.8, 2.85) | 3.12 (3.09, 3.14) |
|  | Final year of primary school (0) | 3.84 (3.82, 3.86) | 3.77 (3.75, 3.78) | 3.61 (3.59, 3.62) | 3.7 (3.69, 3.72) | 3.27 (3.25, 3.29) | 3.6 (3.59, 3.62) | 2.85 (2.83, 2.87) | 3.13 (3.11, 3.15) |
|  | First year post transition (+1) | 3.4 (3.37, 3.42) | 3.61 (3.59, 3.63) | 3.42 (3.39, 3.44) | 3.37 (3.35, 3.39) | 3.11 (3.08, 3.13) | 3.37 (3.35, 3.4) | 2.96 (2.93, 2.99) | 3.19 (3.16, 3.22) |
|  | Second year post transition (+2) | 3.61 (3.58, 3.63) | 3.67 (3.65, 3.69) | 3.5 (3.47, 3.52) | 3.51 (3.49, 3.54) | 3.16 (3.14, 3.19) | 3.48 (3.46, 3.51) | 2.92 (2.89, 2.95) | 3.17 (3.15, 3.2) |
|  | Third year post transition (+3) | 3.4 (3.38, 3.43) | 3.65 (3.63, 3.67) | 3.48 (3.45, 3.5) | 3.39 (3.37, 3.41) | 3.2 (3.17, 3.22) | 3.41 (3.38, 3.43) | 2.93 (2.9, 2.95) | 3.15 (3.12, 3.18) |
|  | Fourth year post transition (+4) | 3.42 (3.39, 3.44) | 3.65 (3.62, 3.67) | 3.49 (3.47, 3.52) | 3.42 (3.39, 3.44) | 3.25 (3.22, 3.28) | 3.41 (3.38, 3.43) | 2.92 (2.89, 2.95) | 3.16 (3.13, 3.19) |
| Inner & outer region | Two years before transition (-2) | 3.99 (3.96, 4.02) | 3.99 (3.96, 4.02) | 3.92 (3.89, 3.95) | 3.84 (3.81, 3.86) | 3.7 (3.67, 3.73) | 3.85 (3.82, 3.88) | 2.63 (2.6, 2.67) | 2.91 (2.87, 2.94) |
|  | One year before transition (-1) | 3.8 (3.77, 3.83) | 3.79 (3.76, 3.82) | 3.67 (3.64, 3.7) | 3.68 (3.65, 3.71) | 3.41 (3.37, 3.44) | 3.66 (3.63, 3.69) | 2.81 (2.78, 2.85) | 3.06 (3.03, 3.1) |
|  | Final year of primary school (0) | 3.81 (3.78, 3.84) | 3.79 (3.77, 3.82) | 3.63 (3.6, 3.66) | 3.71 (3.69, 3.74) | 3.31 (3.28, 3.34) | 3.64 (3.61, 3.66) | 2.82 (2.78, 2.85) | 3.06 (3.03, 3.1) |
|  | First year post transition (+1) | 3.39 (3.36, 3.42) | 3.59 (3.56, 3.62) | 3.42 (3.39, 3.45) | 3.42 (3.4, 3.45) | 3.08 (3.04, 3.11) | 3.38 (3.35, 3.41) | 2.97 (2.93, 3.01) | 3.17 (3.14, 3.21) |
|  | Second year post transition (+2) | 3.63 (3.6, 3.66) | 3.7 (3.67, 3.73) | 3.56 (3.53, 3.6) | 3.59 (3.56, 3.62) | 3.2 (3.17, 3.24) | 3.55 (3.52, 3.58) | 2.89 (2.86, 2.93) | 3.15 (3.12, 3.19) |
|  | Third year post transition (+3) | 3.39 (3.36, 3.43) | 3.63 (3.6, 3.66) | 3.46 (3.42, 3.49) | 3.44 (3.41, 3.47) | 3.14 (3.11, 3.18) | 3.4 (3.36, 3.43) | 2.93 (2.89, 2.97) | 3.17 (3.13, 3.2) |
|  | Fourth year post transition (+4) | 3.4 (3.37, 3.43) | 3.61 (3.58, 3.64) | 3.44 (3.41, 3.48) | 3.45 (3.42, 3.48) | 3.17 (3.14, 3.21) | 3.37 (3.33, 3.4) | 2.96 (2.92, 3) | 3.2 (3.16, 3.24) |
| Remote & very remote region | Two years before transition (-2) | 3.98 (3.9, 4.07) | 3.95 (3.87, 4.03) | 3.91 (3.83, 4) | 3.81 (3.73, 3.88) | 3.7 (3.6, 3.79) | 3.84 (3.75, 3.92) | 2.69 (2.6, 2.79) | 2.99 (2.89, 3.09) |
|  | One year before transition (-1) | 3.82 (3.74, 3.9) | 3.88 (3.81, 3.95) | 3.76 (3.68, 3.84) | 3.73 (3.66, 3.8) | 3.55 (3.46, 3.63) | 3.77 (3.69, 3.85) | 2.8 (2.71, 2.88) | 3.11 (3.02, 3.2) |
|  | Final year of primary school (0) | 3.85 (3.77, 3.92) | 3.89 (3.83, 3.96) | 3.76 (3.68, 3.84) | 3.75 (3.68, 3.82) | 3.48 (3.39, 3.56) | 3.73 (3.65, 3.8) | 2.68 (2.59, 2.76) | 3 (2.91, 3.09) |
|  | First year post transition (+1) | 3.43 (3.35, 3.51) | 3.66 (3.59, 3.73) | 3.49 (3.41, 3.57) | 3.47 (3.4, 3.54) | 3.2 (3.12, 3.29) | 3.45 (3.37, 3.53) | 2.92 (2.83, 3.01) | 3.13 (3.03, 3.22) |
|  | Second year post transition (+2) | 3.65 (3.58, 3.73) | 3.81 (3.74, 3.88) | 3.67 (3.59, 3.75) | 3.61 (3.54, 3.69) | 3.34 (3.25, 3.42) | 3.64 (3.56, 3.72) | 2.85 (2.76, 2.94) | 3.1 (3.01, 3.2) |
|  | Third year post transition (+3) | 3.34 (3.25, 3.42) | 3.56 (3.49, 3.63) | 3.41 (3.33, 3.49) | 3.42 (3.35, 3.5) | 3.14 (3.06, 3.23) | 3.33 (3.24, 3.41) | 3.01 (2.92, 3.1) | 3.22 (3.13, 3.32) |
|  | Fourth year post transition (+4) | 3.3 (3.22, 3.39) | 3.57 (3.49, 3.64) | 3.4 (3.31, 3.49) | 3.46 (3.38, 3.54) | 3.13 (3.04, 3.23) | 3.32 (3.23, 3.41) | 3.03 (2.93, 3.13) | 3.26 (3.15, 3.36) |

Table S10. Marginal mean for eight wellbeing sub-domains over time across highest parental educational level

| **Highest parental educational level** | **Years since transition** | **Cognitive engagement**  **(N = 100088)** | **Happiness**  **(N = 103364)** | **Optimism**  **(N = 102892)** | **Perseverance**  **(N = 103099)** | **Emotional regulation**  **(N = 102414)** | **Satisfaction with life**  **(N = 102796)** | **Sadness**  **(N = 102475)** | **Worry**  **(N = 102338)** |
| --- | --- | --- | --- | --- | --- | --- | --- | --- | --- |
| Bachelor degree or above | Two years before transition (-2) | 4.04 (4.02, 4.07) | 4.02 (4, 4.04) | 3.95 (3.92, 3.97) | 3.88 (3.86, 3.9) | 3.64 (3.61, 3.67) | 3.88 (3.85, 3.91) | 2.46 (2.44, 2.49) | 2.75 (2.72, 2.78) |
|  | One year before transition (-1) | 3.91 (3.89, 3.94) | 3.85 (3.83, 3.87) | 3.73 (3.71, 3.76) | 3.78 (3.76, 3.8) | 3.41 (3.38, 3.44) | 3.7 (3.67, 3.73) | 2.67 (2.64, 2.7) | 3 (2.96, 3.03) |
|  | Final year of primary school (0) | 3.93 (3.91, 3.96) | 3.84 (3.82, 3.86) | 3.72 (3.69, 3.74) | 3.8 (3.78, 3.82) | 3.32 (3.3, 3.35) | 3.7 (3.67, 3.72) | 2.69 (2.66, 2.71) | 3 (2.97, 3.02) |
|  | First year post transition (+1) | 3.55 (3.53, 3.58) | 3.68 (3.65, 3.7) | 3.52 (3.49, 3.54) | 3.53 (3.5, 3.55) | 3.16 (3.13, 3.19) | 3.46 (3.43, 3.49) | 2.86 (2.83, 2.89) | 3.12 (3.09, 3.15) |
|  | Second year post transition (+2) | 3.75 (3.72, 3.78) | 3.75 (3.72, 3.77) | 3.61 (3.58, 3.64) | 3.66 (3.63, 3.68) | 3.21 (3.19, 3.24) | 3.57 (3.55, 3.6) | 2.8 (2.77, 2.83) | 3.09 (3.06, 3.12) |
|  | Third year post transition (+3) | 3.54 (3.51, 3.56) | 3.7 (3.68, 3.73) | 3.56 (3.53, 3.59) | 3.53 (3.51, 3.56) | 3.24 (3.21, 3.27) | 3.48 (3.45, 3.51) | 2.84 (2.81, 2.87) | 3.09 (3.06, 3.12) |
|  | Fourth year post transition (+4) | 3.54 (3.51, 3.57) | 3.71 (3.68, 3.73) | 3.57 (3.55, 3.6) | 3.55 (3.52, 3.57) | 3.32 (3.29, 3.35) | 3.5 (3.47, 3.52) | 2.85 (2.82, 2.88) | 3.1 (3.07, 3.13) |
| Diploma | Two years before transition (-2) | 3.97 (3.95, 3.99) | 3.97 (3.95, 3.99) | 3.89 (3.87, 3.91) | 3.81 (3.79, 3.83) | 3.67 (3.65, 3.69) | 3.83 (3.81, 3.85) | 2.67 (2.64, 2.69) | 2.94 (2.92, 2.96) |
|  | One year before transition (-1) | 3.76 (3.74, 3.78) | 3.76 (3.74, 3.78) | 3.62 (3.6, 3.65) | 3.65 (3.63, 3.67) | 3.37 (3.35, 3.39) | 3.63 (3.61, 3.65) | 2.88 (2.86, 2.91) | 3.14 (3.12, 3.17) |
|  | Final year of primary school (0) | 3.78 (3.76, 3.8) | 3.76 (3.74, 3.77) | 3.57 (3.55, 3.59) | 3.66 (3.64, 3.68) | 3.27 (3.25, 3.29) | 3.59 (3.57, 3.61) | 2.89 (2.87, 2.91) | 3.15 (3.12, 3.17) |
|  | First year post transition (+1) | 3.34 (3.32, 3.36) | 3.58 (3.56, 3.6) | 3.38 (3.35, 3.4) | 3.34 (3.32, 3.37) | 3.07 (3.05, 3.09) | 3.34 (3.32, 3.36) | 3.01 (2.99, 3.04) | 3.22 (3.19, 3.25) |
|  | Second year post transition (+2) | 3.57 (3.55, 3.59) | 3.66 (3.64, 3.68) | 3.48 (3.46, 3.5) | 3.5 (3.48, 3.52) | 3.15 (3.12, 3.17) | 3.48 (3.45, 3.5) | 2.96 (2.94, 2.99) | 3.21 (3.18, 3.23) |
|  | Third year post transition (+3) | 3.35 (3.32, 3.37) | 3.61 (3.59, 3.63) | 3.42 (3.4, 3.45) | 3.37 (3.35, 3.39) | 3.14 (3.12, 3.17) | 3.37 (3.34, 3.39) | 2.98 (2.95, 3.01) | 3.2 (3.18, 3.23) |
|  | Fourth year post transition (+4) | 3.36 (3.34, 3.39) | 3.6 (3.58, 3.63) | 3.43 (3.41, 3.46) | 3.4 (3.37, 3.42) | 3.17 (3.15, 3.2) | 3.35 (3.33, 3.38) | 2.97 (2.94, 3) | 3.21 (3.18, 3.24) |
| Year 12 or less | Two years before transition (-2) | 3.96 (3.93, 3.99) | 3.95 (3.92, 3.98) | 3.86 (3.83, 3.89) | 3.78 (3.76, 3.81) | 3.69 (3.65, 3.72) | 3.82 (3.79, 3.85) | 2.73 (2.7, 2.76) | 3.01 (2.98, 3.05) |
|  | One year before transition (-1) | 3.73 (3.7, 3.76) | 3.74 (3.72, 3.77) | 3.61 (3.57, 3.64) | 3.61 (3.59, 3.64) | 3.39 (3.36, 3.43) | 3.62 (3.59, 3.66) | 2.91 (2.88, 2.94) | 3.16 (3.12, 3.2) |
|  | Final year of primary school (0) | 3.75 (3.72, 3.77) | 3.73 (3.7, 3.75) | 3.56 (3.53, 3.58) | 3.6 (3.58, 3.63) | 3.3 (3.27, 3.34) | 3.55 (3.52, 3.58) | 2.94 (2.91, 2.97) | 3.19 (3.16, 3.22) |
|  | First year post transition (+1) | 3.26 (3.23, 3.29) | 3.54 (3.5, 3.57) | 3.34 (3.31, 3.38) | 3.27 (3.24, 3.3) | 3.08 (3.04, 3.11) | 3.32 (3.28, 3.35) | 3.04 (3, 3.07) | 3.22 (3.18, 3.26) |
|  | Second year post transition (+2) | 3.49 (3.46, 3.52) | 3.62 (3.59, 3.65) | 3.45 (3.41, 3.48) | 3.43 (3.4, 3.46) | 3.17 (3.13, 3.2) | 3.44 (3.41, 3.48) | 3 (2.97, 3.04) | 3.22 (3.18, 3.26) |
|  | Third year post transition (+3) | 3.28 (3.25, 3.32) | 3.6 (3.57, 3.63) | 3.41 (3.38, 3.45) | 3.3 (3.27, 3.34) | 3.15 (3.11, 3.18) | 3.35 (3.32, 3.39) | 2.97 (2.93, 3.01) | 3.18 (3.14, 3.22) |
|  | Fourth year post transition (+4) | 3.28 (3.24, 3.32) | 3.56 (3.53, 3.59) | 3.39 (3.36, 3.43) | 3.31 (3.28, 3.34) | 3.17 (3.13, 3.21) | 3.3 (3.26, 3.34) | 3.02 (2.98, 3.06) | 3.24 (3.2, 3.28) |

Table S11. Marginal effects over time

| **Contrast** | **Cognitive engagement**  **(N = 100088)** | | **Happiness**  **(N = 103364)** | | **Optimism**  **(N = 102892)** | | **Perseverance**  **(N = 103099)** | | **Emotional regulation**  **(N = 102414)** | | **Satisfaction with life**  **(N = 102796)** | | **Sadness**  **(N = 102475)** | | **Worry**  **(N = 102338)** | |
| --- | --- | --- | --- | --- | --- | --- | --- | --- | --- | --- | --- | --- | --- | --- | --- | --- |
|  | Difference (95% CI) | *P* | Difference (95% CI) | *P* | Difference (95% CI) | *P* | Difference (95% CI) | *P* | Difference (95% CI) | *P* | Difference (95% CI) | *P* | Difference (95% CI) | *P* | Difference (95% CI) | *P* |
| Pandemic period (−2~−1) | -0.19 (-0.20, -0.17) | <0.001 | -0.20 (-0.21, -0.18) | <0.001 | -0.25 (-0.26, -0.23) | <0.001 | -0.14 (-0.16, -0.13) | <0.001 | -0.28 (-0.29, -0.26) | <0.001 | -0.19 (-0.21, -0.18) | <0.001 | 0.21 (0.19, 0.22) | <0.001 | 0.21 (0.19, 0.23) | <0.001 |
| Primary school period (−1~0) | 0.02 (0.01, 0.04) | 0.002 | -0.01 (-0.02, 0.00) | 0.162 | -0.04 (-0.05, -0.02) | <0.001 | 0.01 (-0.00, 0.02) | 0.156 | -0.10 (-0.11, -0.08) | <0.001 | -0.04 (-0.05, -0.02) | <0.001 | 0.01 (-0.00, 0.03) | 0.145 | 0.01 (-0.01, 0.03) | 0.46 |
| Transition period  (0~+1) | -0.22 (-0.24, -0.20) | <0.001 | -0.10 (-0.12, -0.08) | <0.001 | -0.10 (-0.12, -0.08) | <0.001 | -0.16 (-0.18, -0.14) | <0.001 | -0.13 (-0.15, -0.10) | <0.001 | -0.12 (-0.14, -0.10) | <0.001 | 0.08 (0.06, 0.10) | <0.001 | 0.06 (0.04, 0.09) | <0.001 |
| Second year post transition period  (+1~+2) | -0.22 (-0.23, -0.20) | <0.001 | -0.08 (-0.09, -0.06) | <0.001 | -0.10 (-0.11, -0.08) | <0.001 | -0.15 (-0.16, -0.14) | <0.001 | -0.07 (-0.09, -0.06) | <0.001 | -0.13 (-0.14, -0.11) | <0.001 | 0.05 (0.03, 0.07) | <0.001 | 0.02 (-0.00, 0.03) | 0.097 |
| Third year post transition period  (+2~+3) | 0.00 (-0.01, 0.02) | 0.91 | 0.04 (0.02, 0.05) | <0.001 | 0.05 (0.03, 0.06) | <0.001 | 0.02 (0.01, 0.03) | 0.006 | 0.08 (0.06, 0.09) | <0.001 | 0.03 (0.01, 0.04) | 0.001 | -0.03 (-0.05, -0.01) | <0.001 | -0.02 (-0.04, -0.01) | 0.008 |
| Fourth year post transition period  (+3~+4) | 0.01 (-0.01, 0.03) | 0.245 | -0.01 (-0.02, 0.01) | 0.217 | 0.01 (-0.01, 0.02) | 0.356 | 0.02 (0.01, 0.03) | 0.005 | 0.05 (0.03, 0.06) | <0.001 | -0.01 (-0.03, 0.01) | 0.229 | 0.00 (-0.02, 0.02) | 0.763 | 0.02 (-0.00, 0.04) | 0.107 |

Table S12. Marginal effects over time across age cohort

| **Contrast** | **Age cohort** | **Cognitive engagement**  **(N = 100088)** | | **Happiness**  **(N = 103364)** | | **Optimism**  **(N = 102892)** | | **Perseverance**  **(N = 103099)** | | **Emotional regulation**  **(N = 102414)** | | **Satisfaction with life**  **(N = 102796)** | | **Sadness**  **(N = 102475)** | | **Worry**  **(N = 102338)** | |
| --- | --- | --- | --- | --- | --- | --- | --- | --- | --- | --- | --- | --- | --- | --- | --- | --- | --- |
|  |  | Difference (95% CI) | *P* | Difference (95% CI) | *P* | Difference (95% CI) | *P* | Difference (95% CI) | *P* | Difference (95% CI) | *P* | Difference (95% CI) | *P* | Difference (95% CI) | *P* | Difference (95% CI) | *P* |
| Pandemic period (−2~−1) | Old | -0.22 (-0.24, -0.20) | <0.001 | -0.21 (-0.23, -0.19) | <0.001 | -0.25 (-0.27, -0.22) | <0.001 | -0.16 (-0.18, -0.14) | <0.001 | -0.27 (-0.30, -0.25) | <0.001 | -0.22 (-0.24, -0.20) | <0.001 | 0.23 (0.20, 0.25) | <0.001 | 0.24 (0.21, 0.27) | <0.001 |
|  | Young | -0.16 (-0.18, -0.14) | <0.001 | -0.18 (-0.20, -0.16) | <0.001 | -0.25 (-0.27, -0.22) | <0.001 | -0.13 (-0.15, -0.11) | <0.001 | -0.28 (-0.31, -0.26) | <0.001 | -0.17 (-0.19, -0.14) | <0.001 | 0.19 (0.16, 0.21) | <0.001 | 0.17 (0.15, 0.20) | <0.001 |
| Primary school period (−1~0) | Old | 0.02 (0.00, 0.04) | 0.049 | -0.02 (-0.04, 0.00) | 0.092 | -0.06 (-0.08, -0.03) | <0.001 | 0.00 (-0.01, 0.02) | 0.675 | -0.09 (-0.12, -0.07) | <0.001 | -0.05 (-0.07, -0.03) | <0.001 | 0.02 (-0.01, 0.04) | 0.137 | 0.01 (-0.02, 0.03) | 0.641 |
|  | Young | 0.03 (0.00, 0.05) | 0.016 | -0.00 (-0.02, 0.02) | 0.763 | -0.02 (-0.04, -0.00) | 0.045 | 0.02 (-0.00, 0.03) | 0.11 | -0.10 (-0.12, -0.07) | <0.001 | -0.02 (-0.05, -0.00) | 0.027 | 0.01 (-0.02, 0.03) | 0.566 | 0.01 (-0.02, 0.03) | 0.576 |
| Transition period  (0~+1) | Old | -0.21 (-0.24, -0.19) | <0.001 | -0.10 (-0.12, -0.08) | <0.001 | -0.09 (-0.11, -0.06) | <0.001 | -0.16 (-0.18, -0.13) | <0.001 | -0.11 (-0.13, -0.08) | <0.001 | -0.11 (-0.14, -0.09) | <0.001 | 0.07 (0.05, 0.10) | <0.001 | 0.05 (0.02, 0.08) | 0.001 |
|  | Young | -0.23 (-0.25, -0.20) | <0.001 | -0.10 (-0.13, -0.08) | <0.001 | -0.12 (-0.15, -0.10) | <0.001 | -0.17 (-0.19, -0.15) | <0.001 | -0.15 (-0.17, -0.12) | <0.001 | -0.12 (-0.14, -0.09) | <0.001 | 0.09 (0.06, 0.12) | <0.001 | 0.08 (0.05, 0.11) | <0.001 |
| Second year post transition period  (+1~+2) | Old | -0.19 (-0.21, -0.17) | <0.001 | -0.07 (-0.08, -0.05) | <0.001 | -0.09 (-0.11, -0.07) | <0.001 | -0.12 (-0.14, -0.10) | <0.001 | -0.04 (-0.07, -0.02) | <0.001 | -0.11 (-0.14, -0.09) | <0.001 | 0.03 (0.01, 0.05) | 0.013 | -0.00 (-0.03, 0.02) | 0.716 |
|  | Young | -0.25 (-0.27, -0.23) | <0.001 | -0.09 (-0.11, -0.07) | <0.001 | -0.11 (-0.13, -0.09) | <0.001 | -0.18 (-0.20, -0.16) | <0.001 | -0.10 (-0.12, -0.08) | <0.001 | -0.14 (-0.16, -0.12) | <0.001 | 0.07 (0.05, 0.09) | <0.001 | 0.03 (0.01, 0.06) | 0.008 |
| Third year post transition period  (+2~+3) | Old | 0.02 (0.00, 0.04) | 0.038 | 0.05 (0.03, 0.07) | <0.001 | 0.08 (0.05, 0.10) | <0.001 | 0.04 (0.02, 0.06) | <0.001 | 0.11 (0.08, 0.13) | <0.001 | 0.06 (0.04, 0.08) | <0.001 | -0.03 (-0.06, -0.01) | 0.014 | 0.01 (-0.02, 0.04) | 0.522 |
|  | Young | -0.02 (-0.04, 0.00) | 0.083 | 0.02 (0.00, 0.04) | 0.044 | 0.02 (0.00, 0.04) | 0.049 | 0.00 (-0.02, 0.02) | 0.865 | 0.04 (0.02, 0.07) | <0.001 | -0.00 (-0.03, 0.02) | 0.662 | -0.03 (-0.06, -0.01) | 0.01 | -0.06 (-0.08, -0.03) | <0.001 |
| Fourth year post transition period  (+3~+4) | Old | 0.01 (-0.01, 0.03) | 0.36 | -0.04 (-0.06, -0.02) | <0.001 | -0.02 (-0.04, 0.01) | 0.15 | -0.00 (-0.02, 0.02) | 0.876 | 0.00 (-0.02, 0.03) | 0.874 | -0.03 (-0.05, -0.01) | 0.014 | 0.01 (-0.01, 0.04) | 0.345 | 0.01 (-0.02, 0.04) | 0.386 |
|  | Young | 0.01 (-0.01, 0.03) | 0.445 | 0.02 (-0.00, 0.04) | 0.1 | 0.03 (0.01, 0.05) | 0.006 | 0.04 (0.02, 0.06) | <0.001 | 0.09 (0.06, 0.11) | <0.001 | 0.01 (-0.01, 0.03) | 0.469 | -0.01 (-0.03, 0.02) | 0.625 | 0.02 (-0.01, 0.05) | 0.154 |

Table S13. Marginal effects over time across gender

| **Contrast** | **Gender** | **Cognitive engagement**  **(N = 100088)** | | **Happiness**  **(N = 103364)** | | **Optimism**  **(N = 102892)** | | **Perseverance**  **(N = 103099)** | | **Emotional regulation**  **(N = 102414)** | | **Satisfaction with life**  **(N = 102796)** | | **Sadness**  **(N = 102475)** | | **Worry**  **(N = 102338)** | |
| --- | --- | --- | --- | --- | --- | --- | --- | --- | --- | --- | --- | --- | --- | --- | --- | --- | --- |
|  |  | Difference (95% CI) | *P* | Difference (95% CI) | *P* | Difference (95% CI) | *P* | Difference (95% CI) | *P* | Difference (95% CI) | *P* | Difference (95% CI) | *P* | Difference (95% CI) | *P* | Difference (95% CI) | *P* |
| Pandemic period (−2~−1) | Female | -0.23 (-0.25, -0.21) | <0.001 | -0.27 (-0.29, -0.25) | <0.001 | -0.34 (-0.37, -0.32) | <0.001 | -0.20 (-0.22, -0.18) | <0.001 | -0.35 (-0.38, -0.33) | <0.001 | -0.27 (-0.30, -0.25) | <0.001 | 0.29 (0.27, 0.32) | <0.001 | 0.31 (0.29, 0.34) | <0.001 |
|  | Male | -0.15 (-0.17, -0.13) | <0.001 | -0.13 (-0.15, -0.11) | <0.001 | -0.15 (-0.18, -0.13) | <0.001 | -0.09 (-0.11, -0.07) | <0.001 | -0.21 (-0.23, -0.18) | <0.001 | -0.12 (-0.14, -0.09) | <0.001 | 0.13 (0.10, 0.15) | <0.001 | 0.11 (0.08, 0.13) | <0.001 |
| Primary school period (−1~0) | Female | 0.01 (-0.01, 0.03) | 0.226 | -0.04 (-0.06, -0.02) | <0.001 | -0.06 (-0.08, -0.04) | <0.001 | -0.00 (-0.02, 0.02) | 0.717 | -0.12 (-0.15, -0.10) | <0.001 | -0.06 (-0.08, -0.04) | <0.001 | 0.05 (0.03, 0.08) | <0.001 | 0.05 (0.02, 0.07) | <0.001 |
|  | Male | 0.03 (0.01, 0.05) | 0.002 | 0.02 (-0.00, 0.04) | 0.07 | -0.02 (-0.04, 0.00) | 0.098 | 0.02 (0.00, 0.04) | 0.014 | -0.07 (-0.09, -0.04) | <0.001 | -0.02 (-0.04, 0.00) | 0.102 | -0.02 (-0.05, -0.00) | 0.04 | -0.03 (-0.06, -0.01) | 0.016 |
| Transition period  (0~+1) | Female | -0.26 (-0.28, -0.24) | <0.001 | -0.15 (-0.17, -0.13) | <0.001 | -0.14 (-0.17, -0.12) | <0.001 | -0.22 (-0.24, -0.20) | <0.001 | -0.18 (-0.20, -0.15) | <0.001 | -0.16 (-0.19, -0.14) | <0.001 | 0.13 (0.11, 0.16) | <0.001 | 0.12 (0.09, 0.15) | <0.001 |
|  | Male | -0.18 (-0.20, -0.15) | <0.001 | -0.05 (-0.07, -0.03) | <0.001 | -0.06 (-0.09, -0.04) | <0.001 | -0.10 (-0.12, -0.08) | <0.001 | -0.07 (-0.10, -0.05) | <0.001 | -0.07 (-0.09, -0.04) | <0.001 | 0.03 (0.00, 0.06) | 0.042 | 0.00 (-0.03, 0.03) | 0.826 |
| Second year post transition period  (+1~+2) | Female | -0.26 (-0.28, -0.24) | <0.001 | -0.11 (-0.13, -0.09) | <0.001 | -0.16 (-0.18, -0.14) | <0.001 | -0.21 (-0.23, -0.19) | <0.001 | -0.13 (-0.15, -0.11) | <0.001 | -0.18 (-0.20, -0.16) | <0.001 | 0.12 (0.09, 0.14) | <0.001 | 0.09 (0.06, 0.11) | <0.001 |
|  | Male | -0.18 (-0.20, -0.16) | <0.001 | -0.04 (-0.06, -0.03) | <0.001 | -0.05 (-0.07, -0.03) | <0.001 | -0.09 (-0.11, -0.08) | <0.001 | -0.02 (-0.05, 0.00) | 0.072 | -0.08 (-0.10, -0.06) | <0.001 | -0.01 (-0.03, 0.01) | 0.474 | -0.05 (-0.07, -0.03) | <0.001 |
| Third year post transition period  (+2~+3) | Female | 0.02 (0.00, 0.04) | 0.039 | 0.06 (0.04, 0.08) | <0.001 | 0.06 (0.04, 0.09) | <0.001 | 0.03 (0.01, 0.05) | 0.005 | 0.12 (0.09, 0.14) | <0.001 | 0.05 (0.03, 0.07) | <0.001 | -0.03 (-0.05, -0.01) | 0.018 | -0.02 (-0.05, 0.01) | 0.136 |
|  | Male | -0.02 (-0.04, 0.00) | 0.067 | 0.02 (-0.00, 0.03) | 0.101 | 0.03 (0.01, 0.05) | 0.004 | 0.01 (-0.01, 0.03) | 0.296 | 0.04 (0.01, 0.06) | 0.003 | 0.00 (-0.02, 0.02) | 0.794 | -0.03 (-0.06, -0.01) | 0.008 | -0.03 (-0.05, -0.00) | 0.024 |
| Fourth year post transition period  (+3~+4) | Female | 0.03 (0.01, 0.05) | 0.016 | 0.01 (-0.01, 0.03) | 0.414 | 0.03 (0.00, 0.05) | 0.018 | 0.05 (0.03, 0.07) | <0.001 | 0.06 (0.04, 0.09) | <0.001 | 0.02 (-0.01, 0.04) | 0.166 | -0.00 (-0.03, 0.02) | 0.799 | 0.01 (-0.02, 0.04) | 0.441 |
|  | Male | -0.01 (-0.03, 0.01) | 0.482 | -0.02 (-0.04, -0.00) | 0.014 | -0.01 (-0.03, 0.01) | 0.347 | -0.00 (-0.02, 0.02) | 0.685 | 0.03 (0.00, 0.05) | 0.033 | -0.03 (-0.06, -0.01) | 0.003 | 0.01 (-0.02, 0.03) | 0.554 | 0.02 (-0.01, 0.05) | 0.151 |

Table S14. Marginal effects over time across main language spoken at home

| **Contrast** | **Primary language spoken at home** | **Cognitive engagement**  **(N = 100088)** | | **Happiness**  **(N = 103364)** | | **Optimism**  **(N = 102892)** | | **Perseverance**  **(N = 103099)** | | **Emotional regulation**  **(N = 102414)** | | **Satisfaction with life**  **(N = 102796)** | | **Sadness**  **(N = 102475)** | | **Worry**  **(N = 102338)** | |
| --- | --- | --- | --- | --- | --- | --- | --- | --- | --- | --- | --- | --- | --- | --- | --- | --- | --- |
|  |  | Difference (95% CI) | *P* | Difference (95% CI) | *P* | Difference (95% CI) | *P* | Difference (95% CI) | *P* | Difference (95% CI) | *P* | Difference (95% CI) | *P* | Difference (95% CI) | *P* | Difference (95% CI) | *P* |
| Pandemic period (−2~−1) | English | -0.21 (-0.23, -0.19) | <0.001 | -0.21 (-0.22, -0.19) | <0.001 | -0.25 (-0.27, -0.23) | <0.001 | -0.16 (-0.17, -0.14) | <0.001 | -0.29 (-0.31, -0.27) | <0.001 | -0.20 (-0.21, -0.18) | <0.001 | 0.22 (0.20, 0.24) | <0.001 | 0.21 (0.19, 0.23) | <0.001 |
|  | LOTE | -0.11 (-0.15, -0.08) | <0.001 | -0.15 (-0.18, -0.12) | <0.001 | -0.22 (-0.26, -0.19) | <0.001 | -0.09 (-0.12, -0.06) | <0.001 | -0.23 (-0.27, -0.19) | <0.001 | -0.18 (-0.22, -0.15) | <0.001 | 0.16 (0.12, 0.20) | <0.001 | 0.20 (0.16, 0.24) | <0.001 |
| Primary school period (−1~0) | English | 0.02 (0.01, 0.04) | 0.004 | -0.00 (-0.02, 0.01) | 0.896 | -0.04 (-0.06, -0.02) | <0.001 | 0.01 (-0.01, 0.02) | 0.263 | -0.10 (-0.12, -0.08) | <0.001 | -0.03 (-0.05, -0.02) | <0.001 | 0.01 (-0.01, 0.03) | 0.494 | 0.01 (-0.01, 0.03) | 0.329 |
|  | LOTE | 0.02 (-0.01, 0.05) | 0.272 | -0.04 (-0.07, -0.01) | 0.007 | -0.05 (-0.08, -0.01) | 0.007 | 0.01 (-0.01, 0.04) | 0.36 | -0.09 (-0.12, -0.05) | <0.001 | -0.05 (-0.08, -0.01) | 0.006 | 0.03 (-0.00, 0.07) | 0.068 | -0.01 (-0.04, 0.03) | 0.781 |
| Transition period  (0~+1) | English | -0.22 (-0.24, -0.19) | <0.001 | -0.10 (-0.12, -0.08) | <0.001 | -0.10 (-0.12, -0.08) | <0.001 | -0.16 (-0.18, -0.14) | <0.001 | -0.12 (-0.14, -0.10) | <0.001 | -0.11 (-0.13, -0.09) | <0.001 | 0.08 (0.06, 0.11) | <0.001 | 0.06 (0.04, 0.09) | <0.001 |
|  | LOTE | -0.20 (-0.23, -0.16) | <0.001 | -0.09 (-0.12, -0.06) | <0.001 | -0.11 (-0.14, -0.07) | <0.001 | -0.15 (-0.18, -0.12) | <0.001 | -0.13 (-0.17, -0.09) | <0.001 | -0.13 (-0.16, -0.09) | <0.001 | 0.07 (0.03, 0.11) | <0.001 | 0.05 (0.01, 0.09) | 0.018 |
| Second year post transition period  (+1~+2) | English | -0.23 (-0.25, -0.22) | <0.001 | -0.09 (-0.11, -0.08) | <0.001 | -0.11 (-0.13, -0.10) | <0.001 | -0.16 (-0.18, -0.15) | <0.001 | -0.09 (-0.11, -0.07) | <0.001 | -0.14 (-0.16, -0.13) | <0.001 | 0.06 (0.04, 0.08) | <0.001 | 0.02 (0.00, 0.04) | 0.041 |
|  | LOTE | -0.17 (-0.20, -0.14) | <0.001 | -0.02 (-0.05, 0.01) | 0.143 | -0.05 (-0.08, -0.02) | 0.002 | -0.10 (-0.13, -0.08) | <0.001 | -0.02 (-0.06, 0.01) | 0.259 | -0.07 (-0.10, -0.04) | <0.001 | 0.02 (-0.01, 0.06) | 0.195 | -0.01 (-0.04, 0.03) | 0.77 |
| Third year post transition period  (+2~+3) | English | -0.01 (-0.02, 0.01) | 0.561 | 0.04 (0.02, 0.05) | <0.001 | 0.05 (0.03, 0.06) | <0.001 | 0.01 (-0.00, 0.03) | 0.058 | 0.07 (0.05, 0.09) | <0.001 | 0.02 (0.00, 0.04) | 0.013 | -0.03 (-0.05, -0.01) | 0.004 | -0.02 (-0.04, -0.00) | 0.037 |
|  | LOTE | 0.02 (-0.01, 0.05) | 0.174 | 0.03 (-0.00, 0.05) | 0.064 | 0.05 (0.02, 0.09) | 0.001 | 0.03 (0.00, 0.06) | 0.023 | 0.09 (0.05, 0.13) | <0.001 | 0.04 (0.00, 0.07) | 0.028 | -0.04 (-0.08, -0.00) | 0.033 | -0.03 (-0.07, 0.00) | 0.086 |
| Fourth year post transition period  (+3~+4) | English | 0.02 (0.00, 0.04) | 0.029 | -0.00 (-0.02, 0.01) | 0.56 | 0.01 (-0.00, 0.03) | 0.131 | 0.03 (0.01, 0.04) | <0.001 | 0.05 (0.03, 0.07) | <0.001 | -0.01 (-0.03, 0.01) | 0.433 | 0.00 (-0.02, 0.02) | 0.999 | 0.01 (-0.01, 0.03) | 0.317 |
|  | LOTE | -0.03 (-0.06, 0.01) | 0.119 | -0.02 (-0.05, 0.01) | 0.134 | -0.01 (-0.05, 0.02) | 0.401 | -0.01 (-0.04, 0.02) | 0.51 | 0.02 (-0.02, 0.06) | 0.271 | -0.02 (-0.05, 0.02) | 0.284 | 0.01 (-0.03, 0.05) | 0.538 | 0.03 (-0.01, 0.07) | 0.127 |

Table S15. Marginal effects over time across residential region

| **Contrast** | **Residential region** | **Cognitive engagement**  **(N = 100088)** | | **Happiness**  **(N = 103364)** | | **Optimism**  **(N = 102892)** | | **Perseverance**  **(N = 103099)** | | **Emotional regulation**  **(N = 102414)** | | **Satisfaction with life**  **(N = 102796)** | | **Sadness**  **(N = 102475)** | | **Worry**  **(N = 102338)** | |
| --- | --- | --- | --- | --- | --- | --- | --- | --- | --- | --- | --- | --- | --- | --- | --- | --- | --- |
|  |  | Difference (95% CI) | *P* | Difference (95% CI) | *P* | Difference (95% CI) | *P* | Difference (95% CI) | *P* | Difference (95% CI) | *P* | Difference (95% CI) | *P* | Difference (95% CI) | *P* | Difference (95% CI) | *P* |
| Pandemic period (−2~−1) | Major city | -0.19 (-0.21, -0.17) | <0.001 | -0.20 (-0.22, -0.18) | <0.001 | -0.25 (-0.27, -0.23) | <0.001 | -0.14 (-0.16, -0.12) | <0.001 | -0.28 (-0.30, -0.25) | <0.001 | -0.20 (-0.22, -0.18) | <0.001 | 0.22 (0.20, 0.24) | <0.001 | 0.23 (0.21, 0.25) | <0.001 |
|  | Inner & outer regional | -0.19 (-0.22, -0.16) | <0.001 | -0.20 (-0.23, -0.18) | <0.001 | -0.25 (-0.28, -0.22) | <0.001 | -0.16 (-0.18, -0.13) | <0.001 | -0.29 (-0.33, -0.26) | <0.001 | -0.20 (-0.23, -0.16) | <0.001 | 0.18 (0.15, 0.22) | <0.001 | 0.16 (0.12, 0.19) | <0.001 |
|  | Remote & very remote | -0.17 (-0.24, -0.09) | <0.001 | -0.07 (-0.14, 0.00) | 0.055 | -0.15 (-0.24, -0.07) | 0.001 | -0.08 (-0.15, -0.01) | 0.03 | -0.15 (-0.25, -0.05) | 0.003 | -0.07 (-0.15, 0.02) | 0.123 | 0.10 (0.01, 0.20) | 0.032 | 0.12 (0.02, 0.22) | 0.02 |
| Primary school period (−1~0) | Major city | 0.03 (0.01, 0.05) | 0.002 | -0.01 (-0.03, 0.00) | 0.069 | -0.04 (-0.06, -0.02) | <0.001 | 0.00 (-0.02, 0.02) | 0.937 | -0.10 (-0.12, -0.07) | <0.001 | -0.04 (-0.06, -0.02) | <0.001 | 0.02 (0.00, 0.04) | 0.026 | 0.01 (-0.01, 0.04) | 0.193 |
|  | Inner & outer regional | 0.01 (-0.02, 0.04) | 0.613 | 0.00 (-0.02, 0.03) | 0.768 | -0.04 (-0.07, -0.01) | 0.013 | 0.03 (0.00, 0.06) | 0.021 | -0.09 (-0.13, -0.06) | <0.001 | -0.02 (-0.05, 0.01) | 0.144 | 0.00 (-0.03, 0.04) | 0.932 | 0.00 (-0.04, 0.04) | 0.956 |
|  | Remote & very remote | 0.03 (-0.04, 0.10) | 0.426 | 0.02 (-0.05, 0.08) | 0.642 | -0.00 (-0.08, 0.08) | 0.971 | 0.02 (-0.04, 0.09) | 0.533 | -0.07 (-0.16, 0.02) | 0.109 | -0.04 (-0.12, 0.03) | 0.27 | -0.12 (-0.20, -0.03) | 0.006 | -0.11 (-0.20, -0.02) | 0.017 |
| Transition period  (0~+1) | Major city | -0.23 (-0.26, -0.21) | <0.001 | -0.10 (-0.12, -0.07) | <0.001 | -0.11 (-0.13, -0.08) | <0.001 | -0.19 (-0.21, -0.17) | <0.001 | -0.11 (-0.14, -0.09) | <0.001 | -0.12 (-0.15, -0.10) | <0.001 | 0.07 (0.04, 0.10) | <0.001 | 0.05 (0.02, 0.07) | 0.003 |
|  | Inner & outer region | -0.18 (-0.21, -0.14) | <0.001 | -0.09 (-0.12, -0.06) | <0.001 | -0.07 (-0.10, -0.03) | <0.001 | -0.12 (-0.15, -0.09) | <0.001 | -0.11 (-0.15, -0.07) | <0.001 | -0.09 (-0.12, -0.05) | <0.001 | 0.08 (0.04, 0.12) | <0.001 | 0.09 (0.05, 0.13) | <0.001 |
|  | Remote & very remote | -0.19 (-0.27, -0.12) | <0.001 | -0.08 (-0.15, -0.01) | 0.021 | -0.09 (-0.17, -0.01) | 0.032 | -0.13 (-0.20, -0.07) | <0.001 | -0.14 (-0.23, -0.05) | 0.003 | -0.09 (-0.17, -0.01) | 0.033 | 0.17 (0.08, 0.26) | <0.001 | 0.10 (0.01, 0.20) | 0.034 |
| Second year post transition period  (+1~+2) | Major city | -0.21 (-0.23, -0.19) | <0.001 | -0.06 (-0.07, -0.04) | <0.001 | -0.08 (-0.10, -0.06) | <0.001 | -0.14 (-0.16, -0.13) | <0.001 | -0.05 (-0.07, -0.03) | <0.001 | -0.11 (-0.13, -0.09) | <0.001 | 0.04 (0.02, 0.06) | <0.001 | 0.01 (-0.01, 0.03) | 0.224 |
|  | Inner & outer regional | -0.24 (-0.27, -0.21) | <0.001 | -0.11 (-0.14, -0.09) | <0.001 | -0.14 (-0.17, -0.11) | <0.001 | -0.16 (-0.19, -0.14) | <0.001 | -0.12 (-0.16, -0.09) | <0.001 | -0.17 (-0.20, -0.14) | <0.001 | 0.08 (0.04, 0.11) | <0.001 | 0.02 (-0.02, 0.06) | 0.265 |
|  | Remote & very remote | -0.23 (-0.30, -0.15) | <0.001 | -0.15 (-0.22, -0.08) | <0.001 | -0.18 (-0.26, -0.10) | <0.001 | -0.15 (-0.21, -0.08) | <0.001 | -0.13 (-0.22, -0.05) | 0.003 | -0.19 (-0.27, -0.11) | <0.001 | 0.07 (-0.02, 0.16) | 0.117 | 0.02 (-0.07, 0.12) | 0.623 |
| Third year post transition period  (+2~+3) | Major city | 0.00 (-0.01, 0.02) | 0.602 | 0.04 (0.02, 0.06) | <0.001 | 0.06 (0.04, 0.08) | <0.001 | 0.02 (0.01, 0.04) | 0.008 | 0.09 (0.07, 0.11) | <0.001 | 0.04 (0.02, 0.05) | <0.001 | -0.03 (-0.05, -0.01) | 0.001 | -0.04 (-0.06, -0.01) | 0.001 |
|  | Inner & outer regional | 0.00 (-0.03, 0.03) | 0.812 | 0.04 (0.02, 0.07) | 0.002 | 0.04 (0.01, 0.07) | 0.018 | 0.02 (-0.01, 0.05) | 0.137 | 0.06 (0.03, 0.10) | <0.001 | 0.02 (-0.01, 0.05) | 0.31 | -0.04 (-0.08, -0.01) | 0.017 | -0.01 (-0.05, 0.03) | 0.627 |
|  | Remote & very remote | -0.09 (-0.17, -0.01) | 0.022 | -0.10 (-0.17, -0.03) | 0.005 | -0.08 (-0.17, -0.00) | 0.044 | -0.05 (-0.11, 0.02) | 0.197 | -0.06 (-0.15, 0.03) | 0.213 | -0.12 (-0.20, -0.04) | 0.003 | 0.09 (0.00, 0.18) | 0.048 | 0.10 (-0.00, 0.19) | 0.052 |
| Fourth year post transition period  (+3~+4) | Major city | 0.01 (-0.01, 0.03) | 0.16 | -0.00 (-0.02, 0.01) | 0.625 | 0.02 (-0.00, 0.03) | 0.126 | 0.03 (0.01, 0.04) | 0.003 | 0.05 (0.03, 0.07) | <0.001 | -0.00 (-0.02, 0.02) | 0.776 | -0.01 (-0.03, 0.02) | 0.54 | 0.01 (-0.01, 0.03) | 0.485 |
|  | Inner & outer regional | 0.00 (-0.03, 0.03) | 0.82 | -0.03 (-0.05, 0.00) | 0.074 | -0.01 (-0.04, 0.02) | 0.481 | 0.00 (-0.03, 0.03) | 0.891 | 0.03 (-0.01, 0.07) | 0.115 | -0.03 (-0.06, 0.00) | 0.05 | 0.03 (-0.01, 0.06) | 0.122 | 0.04 (-0.00, 0.07) | 0.07 |
|  | Remote & very remote | -0.03 (-0.12, 0.05) | 0.423 | 0.01 (-0.07, 0.08) | 0.886 | -0.01 (-0.10, 0.08) | 0.775 | 0.04 (-0.04, 0.11) | 0.319 | -0.01 (-0.11, 0.09) | 0.842 | -0.00 (-0.09, 0.08) | 0.938 | 0.02 (-0.08, 0.12) | 0.745 | 0.03 (-0.07, 0.14) | 0.547 |

Table S16. Marginal effects over time across parental highest education

| **Contrast** | **Parental highest education** | **Cognitive engagement**  **(N = 100088)** | | **Happiness**  **(N = 103364)** | | **Optimism**  **(N = 102892)** | | **Perseverance**  **(N = 103099)** | | **Emotional regulation**  **(N = 102414)** | | **Satisfaction with life**  **(N = 102796)** | | **Sadness**  **(N = 102475)** | | **Worry**  **(N = 102338)** | |
| --- | --- | --- | --- | --- | --- | --- | --- | --- | --- | --- | --- | --- | --- | --- | --- | --- | --- |
|  |  | Difference (95% CI) | *P* | Difference (95% CI) | *P* | Difference (95% CI) | *P* | Difference (95% CI) | *P* | Difference (95% CI) | *P* | Difference (95% CI) | *P* | Difference (95% CI) | *P* | Difference (95% CI) | *P* |
| Pandemic period (−2~−1) | Year 12 or less | -0.23 (-0.27, -0.20) | <0.001 | -0.21 (-0.24, -0.18) | <0.001 | -0.26 (-0.29, -0.22) | <0.001 | -0.17 (-0.20, -0.14) | <0.001 | -0.29 (-0.33, -0.25) | <0.001 | -0.20 (-0.23, -0.16) | <0.001 | 0.18 (0.14, 0.22) | <0.001 | 0.15 (0.11, 0.19) | <0.001 |
|  | Diploma | -0.21 (-0.24, -0.19) | <0.001 | -0.21 (-0.23, -0.19) | <0.001 | -0.26 (-0.29, -0.24) | <0.001 | -0.16 (-0.18, -0.14) | <0.001 | -0.30 (-0.33, -0.27) | <0.001 | -0.20 (-0.23, -0.18) | <0.001 | 0.22 (0.19, 0.24) | <0.001 | 0.20 (0.18, 0.23) | <0.001 |
|  | Bachelor degree or above | -0.13 (-0.16, -0.10) | <0.001 | -0.17 (-0.20, -0.15) | <0.001 | -0.21 (-0.24, -0.18) | <0.001 | -0.10 (-0.12, -0.08) | <0.001 | -0.23 (-0.27, -0.20) | <0.001 | -0.18 (-0.21, -0.15) | <0.001 | 0.21 (0.18, 0.24) | <0.001 | 0.25 (0.21, 0.28) | <0.001 |
| Primary school period (−1~0) | Year 12 or less | 0.02 (-0.01, 0.05) | 0.193 | -0.02 (-0.05, 0.01) | 0.268 | -0.05 (-0.09, -0.02) | 0.004 | -0.01 (-0.04, 0.02) | 0.515 | -0.09 (-0.13, -0.05) | <0.001 | -0.07 (-0.11, -0.04) | <0.001 | 0.03 (-0.01, 0.07) | 0.135 | 0.03 (-0.01, 0.07) | 0.142 |
|  | Diploma | 0.02 (0.00, 0.04) | 0.026 | -0.01 (-0.03, 0.01) | 0.466 | -0.05 (-0.07, -0.03) | <0.001 | 0.01 (-0.01, 0.03) | 0.408 | -0.10 (-0.13, -0.08) | <0.001 | -0.04 (-0.07, -0.02) | <0.001 | 0.01 (-0.02, 0.03) | 0.539 | 0.00 (-0.02, 0.03) | 0.872 |
|  | Bachelor degree or above | 0.02 (-0.00, 0.05) | 0.09 | -0.01 (-0.03, 0.01) | 0.469 | -0.02 (-0.04, 0.01) | 0.244 | 0.02 (0.00, 0.05) | 0.044 | -0.08 (-0.12, -0.05) | <0.001 | -0.01 (-0.03, 0.02) | 0.63 | 0.01 (-0.02, 0.04) | 0.463 | 0.00 (-0.03, 0.03) | 0.988 |
| Transition period  (0~+1) | Year 12 or less | -0.26 (-0.29, -0.22) | <0.001 | -0.10 (-0.14, -0.07) | <0.001 | -0.11 (-0.15, -0.07) | <0.001 | -0.17 (-0.21, -0.14) | <0.001 | -0.14 (-0.18, -0.10) | <0.001 | -0.11 (-0.14, -0.07) | <0.001 | 0.06 (0.02, 0.11) | 0.002 | 0.03 (-0.01, 0.08) | 0.157 |
|  | Diploma | -0.21 (-0.24, -0.19) | <0.001 | -0.10 (-0.12, -0.08) | <0.001 | -0.09 (-0.12, -0.07) | <0.001 | -0.16 (-0.18, -0.14) | <0.001 | -0.12 (-0.15, -0.09) | <0.001 | -0.11 (-0.14, -0.09) | <0.001 | 0.07 (0.05, 0.10) | <0.001 | 0.06 (0.03, 0.09) | <0.001 |
|  | Bachelor degree or above | -0.18 (-0.21, -0.16) | <0.001 | -0.09 (-0.12, -0.07) | <0.001 | -0.11 (-0.14, -0.08) | <0.001 | -0.14 (-0.17, -0.12) | <0.001 | -0.11 (-0.14, -0.08) | <0.001 | -0.12 (-0.15, -0.09) | <0.001 | 0.12 (0.08, 0.15) | <0.001 | 0.10 (0.06, 0.13) | <0.001 |
| Second year post transition period  (+1~+2) | Year 12 or less | -0.23 (-0.27, -0.20) | <0.001 | -0.09 (-0.12, -0.06) | <0.001 | -0.10 (-0.14, -0.07) | <0.001 | -0.16 (-0.19, -0.13) | <0.001 | -0.09 (-0.13, -0.05) | <0.001 | -0.13 (-0.16, -0.09) | <0.001 | 0.03 (-0.01, 0.07) | 0.125 | -0.01 (-0.05, 0.04) | 0.771 |
|  | Diploma | -0.23 (-0.25, -0.21) | <0.001 | -0.08 (-0.10, -0.06) | <0.001 | -0.10 (-0.12, -0.08) | <0.001 | -0.16 (-0.18, -0.14) | <0.001 | -0.08 (-0.10, -0.05) | <0.001 | -0.14 (-0.16, -0.12) | <0.001 | 0.05 (0.03, 0.07) | <0.001 | 0.01 (-0.01, 0.04) | 0.311 |
|  | Bachelor degree or above | -0.19 (-0.22, -0.17) | <0.001 | -0.07 (-0.09, -0.05) | <0.001 | -0.09 (-0.12, -0.07) | <0.001 | -0.13 (-0.15, -0.11) | <0.001 | -0.05 (-0.08, -0.03) | <0.001 | -0.11 (-0.14, -0.09) | <0.001 | 0.06 (0.03, 0.09) | <0.001 | 0.03 (-0.00, 0.06) | 0.077 |
| Third year post transition period  (+2~+3) | Year 12 or less | 0.02 (-0.02, 0.06) | 0.255 | 0.07 (0.03, 0.10) | <0.001 | 0.07 (0.03, 0.11) | <0.001 | 0.04 (0.00, 0.07) | 0.027 | 0.07 (0.03, 0.12) | 0.002 | 0.04 (-0.00, 0.08) | 0.066 | -0.06 (-0.11, -0.02) | 0.004 | -0.04 (-0.08, 0.01) | 0.123 |
|  | Diploma | 0.01 (-0.01, 0.03) | 0.401 | 0.03 (0.01, 0.05) | 0.001 | 0.04 (0.02, 0.07) | <0.001 | 0.02 (0.00, 0.04) | 0.017 | 0.07 (0.05, 0.10) | <0.001 | 0.03 (0.00, 0.05) | 0.022 | -0.03 (-0.06, -0.01) | 0.009 | -0.02 (-0.04, 0.01) | 0.199 |
|  | Bachelor degree or above | -0.02 (-0.04, 0.00) | 0.115 | 0.03 (0.01, 0.05) | 0.016 | 0.04 (0.02, 0.07) | 0.001 | 0.00 (-0.02, 0.03) | 0.758 | 0.08 (0.05, 0.11) | <0.001 | 0.02 (-0.01, 0.05) | 0.133 | -0.01 (-0.04, 0.01) | 0.321 | -0.03 (-0.06, 0.00) | 0.052 |
| Fourth year post transition period  (+3~+4) | Year 12 or less | -0.00 (-0.04, 0.04) | 0.941 | -0.04 (-0.08, -0.01) | 0.017 | -0.02 (-0.06, 0.02) | 0.341 | 0.01 (-0.03, 0.04) | 0.731 | 0.02 (-0.02, 0.07) | 0.322 | -0.05 (-0.10, -0.01) | 0.009 | 0.05 (-0.00, 0.09) | 0.053 | 0.06 (0.01, 0.11) | 0.014 |
|  | Diploma | 0.01 (-0.01, 0.04) | 0.218 | -0.01 (-0.03, 0.01) | 0.51 | 0.01 (-0.01, 0.04) | 0.304 | 0.03 (0.01, 0.05) | 0.005 | 0.03 (0.00, 0.06) | 0.028 | -0.01 (-0.04, 0.01) | 0.257 | -0.01 (-0.04, 0.01) | 0.328 | 0.00 (-0.02, 0.03) | 0.807 |
|  | Bachelor degree or above | 0.01 (-0.02, 0.03) | 0.57 | 0.00 (-0.02, 0.03) | 0.785 | 0.01 (-0.01, 0.04) | 0.333 | 0.01 (-0.01, 0.04) | 0.227 | 0.07 (0.04, 0.11) | <0.001 | 0.01 (-0.01, 0.04) | 0.302 | 0.01 (-0.02, 0.04) | 0.712 | 0.01 (-0.02, 0.05) | 0.429 |
